# A biomimetic semisynthesis enables structural elucidation of selaginellin U: a tautomeric cyclic alkynylphenol from *Selaginella tamariscina*

**DOI:** 10.1098/rsos.170352

**Published:** 2017-07-19

**Authors:** Qin-Feng Zhu, Ying Bao, Zhi-Jun Zhang, Jia Su, Li-Dong Shao, Qin-Shi Zhao

**Affiliations:** 1 State Key Laboratory of Phytochemistry and Plant Resources in West China, Kunming Institute of Botany, Chinese Academy of Sciences, Kunming 650201, People's Republic of China; 2 University of Chinese Academy of Sciences, Beijing 100049, People's Republic of China

**Keywords:** biomimetic semisynthesis, structural elucidation, selaginellins, alkynylphenol, *Selaginella tamariscina*

## Abstract

Two new lactone-containing selaginellins T and U (**1** and **2**) together with eleven known selaginellin derivatives (**3** and **7**–**16**) were isolated from the whole plant of *Selaginella tamariscina*. The structure of tautomeric selaginellin U along with its biogenetic pathway was confirmed and proposed by a cross-validation of the semisynthesis of compound **4** from selaginellin (**3**) and derivation from **2** to **4**. Additionally, compounds **3**, **13** and **16** exhibited good inhibitory activities against β-site amyloid precursor protein cleaving enzyme 1 (BACE1) with IC_50_ values of 81.17, 51.13 and 48.89 µM, respectively.

## 1. Introduction

*Selaginella tamariscina*, a qualified species listed in the Chinese Pharmacopoeia, is abundant with bioactive biflavones, and has been used in traditional Chinese medicine for the treatment of amenorrhoea, dysmenorrhoea and traumatic injury [1]. Selaginellin derivatives, a rare family of natural pigments, are characterized by their acetylenic unit and *p*-quinone methide functionalities. However, these alkynylphenol components from this genus were still unknown until Cheng and colleagues first reported a tautomeric alkynylphenol from *Selaginella sinensis* [2]. Later, several selaginellins and their homologues with various bioactivities, including antibacterial activity [3], antifungal activity [4] and cytotoxicity [5–7], were identified. Recently, Yin and colleagues reported that 6/5/6 ring fused alkynylphenol selaginpulvilins A–D could strongly inhibit phosphodiesterase-4 (PDE4), which is a drug target for the treatment of asthma and chronic obstructive pulmonary disease [8]. Woo and colleagues found that selaginpulvilins A and D could act as protein tyrosine phosphatase 1B (PTP1B) inhibitor for the treatment of type 2 diabetes [9,10].

**Figure 1.**
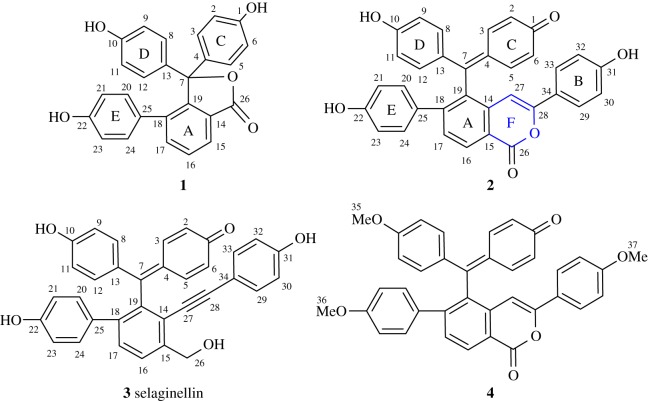
Structures of new compounds **1, 2** and homologues.

Inspired by the structural complexity and diverse bioactivities of selaginellin derivatives, herein, our chemical investigation of *S. tamariscina* led to the isolation and elucidation of two new lactone-containing selaginellins. (During the preparation of this manuscript, Yin and colleagues published the isolation and total synthesis of several lactone-containing selaginellins (see ref. [11]) including selaginpulvilin E, which was a new lactone before the publication), selaginellin T and U (**1** and **2,** figure 1), along with 11 known selaginellin derivatives. Meanwhile, we proposed a biosynthetic pathway for **1** and **2** (electronic supplementary material, scheme S1), as well as other selaginellins isolated from *S. tamariscina* in this study. As shown in scheme S1, oxidation cleavage of the alkynyl group of selaginellin A probably resulted in intermediate **I,** which underwent hydration followed by esterification to yield selaginellin T (**1,** figure 1). Selaginellin (**3,** figure 1) was oxidized to form the aldehyde (selaginellin O), which was further oxidized to give acid. Finally, F-ring closure between C-26 and C-28 and subsequent dehydration yielded selaginellin U (**2,** figure 1). In this work, the structure of compound **2** was determined by a cross-validation of biomimetic semisynthesis from **3** and derivation from **2** to the same product **4**. As a result, the biogenetic hypothesis was validated by this facile synthesis.

## 2. Material and methods

### 2.1. General experimental procedures

UV spectra were measured using a Shimadzu UV-2401A spectrophotometer (Shimadzu, Tokyo, Japan). IR spectra were recorded on a Bruker Tensor 27 spectrophotometer (Bruker Optics, Ettlingen, Germany) with KBr pellets. 1D and 2D NMR spectra were carried out on Bruker AM-400 and DRX-500 or AVANCE III-600 and AV 800 spectrometers with TMS as an internal standard (Bruker, Karlsruhe, Germany). ESI-MS was run on an Agilent 6540 Q TOF spectrometer (Agilent, Palo Alto, CA, USA). HR-ESI-MS was performed using an Agilent G6230 TOF MS system (Agilent, Palo Alto, CA, USA). Semi-preparative HPLC was performed on an Agilent 1260 apparatus equipped with a diode-array detector and a Zorbax SB-C-18 (Agilent, 9.4 mm × 25 cm) column. Column chromatography was performed using MCI gel (CHP 20P, 75–150 mm; Mitsubishi Chemical Corporation, Tokyo, Japan), silica gel (100–200 or 200–300 mesh; Qingdao Marine Chemical Co. Ltd, Qingdao, China) and Sephadex LH-20 (GE Healthcare Bio-Sciences AB, Sala, Sweden). Thin-layer chromatography (TLC) was carried out on silica gel GF254 on glass plates (Qingdao Marine Chemical Inc.) and spots were visualized by heating silica gel plates sprayed with 10% H_2_SO_4_ in EtOH. All reactions sensitive to air or moisture were carried out under an argon or nitrogen atmosphere in dry and freshly distilled solvents under anhydrous conditions, unless otherwise noted.

### 2.2. Plant material

The entire plants of *Selaginella tamariscina* used in this study were purchased from Luosiwan market, Kunming, Yunnan Province, People's Republic of China in 2014 and identified by Prof. Xiao Cheng of the Kunming Institute of Botany, Chinese Academy of Sciences. A voucher specimen (No.2014o608P01) has been deposited at the state key Laboratory of Phytochemistry and Plant Resources in west China.

### 2.3. Extraction and isolation

The air-dried powder of the entire plants of *Selaginella tamariscina* (11 kg) was extracted three times with 70% EtOH (35 l × 3) at room temperature for 72 h, which was then concentrated *in vacuo* to give the deposition portion (450 g). The depositions were subjected to reversed-phase MPLC (MCI; MeOH/H_2_O, 5%–95%, v/v) to give fractions A–E. Fraction C (60 g) was chromatographed over a silica gel column (CHCl_3_/MeOH, 100 : 1–0 : 1) to get five fractions (I–V), based on their TLC characteristics. Fraction III was further separated by use of a silica gel column eluted with CHCl_3_/MeOH (20 : 1–0 : 1, v/v) to give fractions (III-A to III-E). Fraction III-D was further separated by use of a Sephadex LH-20 column (MeOH) and then separated by semi-preparative HPLC (35% MeCN/H_2_O, v/v) to obtain compounds **3** (200 mg), **7** (25 mg), **10** (65 mg) and **13** (5 mg). Fraction III-C was subjected to silica gel column chromatography (petroleum ether/acetone, 6 : 4, v/v) to give two subfractions (III-Ca and III-Cb); III-Ca was separated by semi-preparative HPLC to yield compounds **1** (8 mg), **2** (5 mg), **11** (5 mg) and **14** (3 mg). Subfraction III-Cb was isolated repeatedly by use of a Sephadex LH-20 gel column (MeOH) and then separated by semi-preparative HPLC to yield compounds **8** (8 mg), **9** (18 mg), **12** (37 mg), **15** (10 mg) and **16** (3 mg).

**Selaginellin U (1)**: Off-white powder; UV (CH_3_OH) λmax (log ε) 275 (3.88), 229 (4.46), 203 (4.81) nm; IR (KBr) νmax 3423, 1728, 1612, 1514, 1175 and 838 cm^−1^; ^1^H and ^13^CNMR spectra data; see tables 1 and 2; ESI-MS *m/z*: 409 [M − H]^−^; HR-ESI-MS *m/z*: 433.1047 [M + Na]^+^ (calcd for C_26_H_18_O_5_, 433.1046).

**Table 1.** ^1^H-NMR spectroscopic data for compounds **1**, **2** and **4** (acetone-d_6_).

no.	1^a^	2^b^	4 (route1)^b^ and Δ*δ*^b,c^
2	6.72 (1H, d, 8.7)	*	6.27 (1H, dd, 10.0, 2.7), 0
3	6.89 (1H, d, 8.7)	*	7.23 (1H, dd, 10.0, 2.7), 0
5	6.89 (1H, d, 8.7)	*	7.59 (1H, dd, 10.0, 2.7), 0
6	6.72 (1H, d, 8.7)	*	6.38 (1H, dd, 10.0, 2.7), 0
8/12	6.89 (2H, d, 8.7)	*	6.97 (2H, d, 8.8), 0
9/11	6.72 (2H, d, 8.7)	*	6.82 (2H, d, 8.8), 0
15	7.92 (1H, d, 7.5)	—	—
16	7.71 (1H, t, 7.5)	8.36 (1H, d, 8.1)	8.37 (1H, d, 8.1), 0.01
17	7.52 (1H, d, 7.5)	7.54 (1H, d, 8.1)	7.56 (1H, d, 8.1), 0
20/24	6.54 (2H, d, 8.5)	6.97 (2H, d, 8.4)	7.04 (2H, d, 8.7), 0
21/23	6.58 (2H, d, 8.5)	6.69 (2H, d, 8.4)	6.80 (2H, d, 8.7), 0
27	—	6.71 (1H, s)	6.78 (1H, s), 0
29/33	—	7.58 (2H, d, 8.5)	7.67 (2H, d, 9.0), 0
30/32	—	6.88 (2H, d, 8.5)	6.99 (2H, d, 9.0), 0
35	—	—	3.76 (3H, s), 0
36	—	—	3.78 (3H, s), 0
37	—	—	3.82 (3H, s), 0.01

^a^600 MHz, *δ* in ppm, *J* in Hz.

^b^800 MHz.

*^c^*Δ*δ* is the value of *δ*(**4** from route 1) *−* *δ*(**4** from route 2).

*Ill-defined signals.

**Table 2.** ^13^C-NMR spectroscopic data of **1**, **2** and **4** (*δ* in ppm).

no.	1^a^	2^b^	4(route1)^b,c^	Δ*δ*^b,c^
1	158.4 s	*	186.5 s	0
2	115.4 d	*	129.7 d	0
3	130.9 d	*	138.5 d	0
4	131.4 s	*	132.4 s	0
5	130.9 d	*	139.5 d	0
6	115.4 d	*	129.6 d	0
7	93.9 s	156.0 s	155.1 s	0
8/12	130.9 d	*	133.8 d	0
9/11	115.4 d	*	114.5 d	0
10	158.4 s	*	162.0 s	0
13	131.4 s	*	132.0 s	0
14	127.5 s	138.1 s	137.9 s	0
15	125.0 d	119.7 s	112.0 s	0
16	130.7 d	130.9 d	131.0 d	0
17	138.3 d	130.6 d	130.7 d	0
18	140.5 s	149.7 s	149.4 s	0
19	151.1 s	136.0 s	136.1 s	0
20/24	131.6 d	130.7 d	130.6 d	−0.1
21/23	115.2 d	115.8 d	114.4 d	0
22	157.8 s	158.2 s	160.5 s	0
25	130.5 s	131.9 s	133.0 s	0
26	170.0 s	162.2 s	162.1 s	0
27	—	98.1 d	98.6 d	0
28	—	155.6 s	155.4 s	0
29/33	—	127.8 d	127.7 d	0
30/32	—	116.6 d	115.2 d	0
31	—	160.4 s	162.4 s	0
34	—	124.2 s	125.2 s	0
35	—	—	55.6 q	0
36	—	—	55.0 q	0
37	—	—	55.8 q	0

^a^Measured in acetone-d_6_ at 150 MHz.

^b^Measured in acetone-d_6_ at 200 MHz.

^c^Δ*δ* is the value of *δ*(**4** from route 1) *−* *δ*(**4** from route 2).

*Ill-defined signals.

**Selaginellin T (2)**: yellow oil; UV (CH_3_OH) λmax (log ε) 204 (4.61), 309 (4.36), 357 (4.12), 423 (2.86), 578 (3.40) nm; IR (KBr) νmax 3398, 1704, 1602, 1449, 1167 and 830 cm^−1^; ^1^H and ^13^CNMR spectra data; see tables 1 and 2; ESI-MS *m/z*: 525 [M − H]^−^; HR-ESI-MS *m/z*: 525.1337 [M − H]^−^ (calcd for C_34_H_22_O_6_, 525.1344).

### 2.4. Semisynthesis

**Aldehyde (5).** To a stirred solution of **3** (50 mg) in acetone (20 ml), K_2_CO_3_ (61 mg) and MeI (10 ml) were added. The mixture was sealed and stirred at 50°C for 15 h, and the solvent was evaporated under vacuum. The residue was purified by flash column chromatography on silica gel (CH_3_Cl/MeOH = 20 : 1, v/v) to afford trimethyl ether (50 mg). This compound (50 mg) was dissolved in DCM (25 ml) and to this solution was added activated MnO_2_ (79 mg); the resulting mixture was stirred at 40°C for 24 h. The mixture was filtered and evaporated under vacuum, and the residue was purified by flash column chromatography on silica gel (CH_3_Cl/MeOH = 30 : 1, v/v) to give aldehyde **5** (48 mg, 90% yield over 2 steps). ^1^H-NMR (acetone-d_6_, 600 MHz) δH 8.05 (1H, d, *J* = 8.0 Hz), 7.56 (1H, dd, *J* = 10.0, 2.5 Hz), 7.53 (1H, d, *J* = 8.0 Hz), 7.42 (1H, dd, *J* = 10.0, 2.5 Hz), 7.27 (2H, d, *J* = 8.8 Hz), 7.01 (2H, d, *J* = 8.8 Hz), 6.90 (2H, d, *J* = 8.8 Hz), 6.86 (2H, d, *J* = 8.8 Hz), 6.79 (2H, d, *J* = 8.8 Hz), 6.78 (2H, d, *J* = 8.8 Hz), 6.39 (1H, dd, *J* = 10.0, 2.0 Hz), 6.34 (1H, dd, *J* = 10.0, 2.0 Hz), 3.78 (3H, s, OMe), 3.76 (6H, s, OMe); ^13^C-NMR (acetone-d_6_, 150 MHz) δC 191.7, 186.5, 161.8, 161.7, 160.6, 156.5, 148.6, 143.3, 140.0, 138.7, 135.2, 134.1, 134.1, 133.5, 133.5, 132.6, 132.6, 131.4, 131.1, 130.6, 130.6, 129.6, 129.5, 128.5, 128.3, 115.1, 115.1, 114.6, 114.4, 114.4, 114.3, 114.3, 101.5, 83.6, 55.7, 55.7, 55.6; ESI-MS *m/z*: 553 [M + H]^+^; HR-ESI-MS *m/z*: 575.1862 [M + Na]^+^ (calcd for C_37_H_28_O_5_, 577.1829).

**Acid (6).** To a stirred solution of **5** (45 mg) in a mixture of THF/H_2_O/nBuOH (20 ml, 4 : 4 : 1, v/v) was added 2-methyl-2-butene (0.5 ml), NaH_2_PO_4_ (80 mg) and NaClO_2_ (37 mg). The resulting mixture was stirred at room temperature for 8 h, and the solvent was evaporated under vacuum. The residue was dissolved in H_2_O and extracted with EtOAc (10 ml × 3), dried over Na_2_SO_4_(s). The solvent was evaporated under vacuum and the residue was purified by flash column chromatography on silica gel (petroleum ether/acetone = 4 : 1, v/v) to afford acid **6** (41 mg, 90% yield). ^1^H-NMR(DMSO-d_6_, 800 MHz) δH 7.88 (1H, d, *J* = 8.5 Hz), 7.44 (1H, dd, *J* = 10.0, 2.5 Hz), 7.33 (1H, brd), 7.25 (1H, dd, *J* = 10.0, 2.5 Hz), 7.03 (2H, d, *J* = 8.5 Hz), 6.89 (2H, d, *J* = 8.5 Hz), 6.83 (2H, d, *J* = 8.5 Hz), 6.82 (4H, brs), 6.77 (2H, d, *J* = 8.5 Hz), 6.36 (1H, dd, *J* = 10.0, 2.0 Hz), 6.33 (1H, dd, *J* = 10.0, 2.0 Hz), 3.71 (6H, s, OMe), 3.68 (3H, s, OMe) ^13^C-NMR (DMSO-d_6_, 200 MHz) δC 185.7, 160.4, 159.4, 158.6, 158.1, 141.4, 139.5, 138.2, 132.7, 132.7, 132.5, 132.5, 131.9, 131.2, 130.8, 130.4, 129.8, 129.6, 129.6, 129.3, 128.3, 128.1, 126.3, 125.8, 123.4, 122.4, 114.2, 114.2, 113.6, 113.6, 113.5, 113.5, 97.1, 87.2, 55.4, 55.3, 55.2; ESI-MS *m/z*: 567 [M − H]^−^; HR-ESI-MS *m/z*: 567.1801 [M − H]^−^ (calcd for C_37_H_28_O_6_, 567.1813).

**Lactone (4) prepared from route 1.** To a stirred solution of **6** (38 mg) in MeCN (20 ml), AgNO_3_ (5 mg) was added. The mixture was stirred for 4 h at room temperature and the solvent was evaporated under vacuum. The residue was diluted with H_2_O and extracted with EtOAc (10 ml × 3), dried over Na_2_SO_4_(s). The solvent was evaporated under vacuum and the residue was purified by flash column chromatography on silica gel (petroleum ether/acetone = 5 : 1, v/v) to afford lactone **4** (34 mg, 90% yield). For NMR data, see tables 1 and 2; HR-ESI-MS *m/z*: 607.1525 [M + K]^+^ (calcd for C_37_H_28_O_6_, 607.1517).

**Lactone (4) prepared from route 2.** To a stirred solution of **2** (2 mg) in acetone (3 ml), K_2_CO_3_ (2.2 mg) and MeI (2 ml) were added. The mixture was sealed and stirred at 50°C for 15 h, and the solvent was evaporated under vacuum. The residue was purified by flash column chromatography on silica gel (CH_3_Cl/MeOH = 20 : 1, v/v) to afford lactone **4** (2 mg) in quantitative yield. For NMR data, see tables 1 and 2; HR-ESI-MS *m/z*: 607.1525 [M + K]^+^ (calcd for C_37_H_28_O_6_, 607.1517).

### 2.5. Bioassay for BACE1 inhibitory activity

Compounds **1**–**3** and **7**–**16** were tested for β-site amyloid precursor protein (APP) cleaving enzyme 1 (BACE1) inhibitory activity. BACE1 inhibitory evaluation was performed with a fluorescence resonance energy transfer (FRET) assay kit supplied by Sigma (Kit CS0010, St. Louis, USA). The kit used purified baculovirus-expressed BACE1 and FRET peptide substrates. The first orally available non-peptidic β-secretase inhibitor LY2811376, which had an IC_50_ value of 392 nM, was used as a positive control.

## 3. Results and discussion

**Figure 2.**
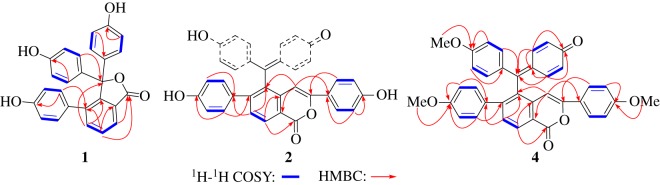
^1^H–^1^H COSY, HMBC and ROESY correlations of compounds **1**, **2** and **4.**

Selaginellin T (**1**) was obtained as an off-white powder. Its molecular formula was determined as C_26_H_18_O_5_ by HR-ESI-MS with an ion peak at *m/z* 433.1047 [M + Na]^+^ (calcd 433.1046), which indicated 18 degrees of unsaturation. The ^1^H-NMR spectrum showed signals for three *p*-phenyl groups (two were overlapped) (*δ*_H_ 6.89 (4H, d, *J* = 8.7 Hz), 6.72 (4H, d, *J* = 8.7 Hz), 6.54 (2H, d, *J* = 8.5 Hz), 6.58 (2H, d, *J* = 8.5 Hz)), and a 1,2,3-trisubstituted benzene ring (*δ*_H_ 7.92 (1H, d, *J* = 7.5 Hz), 7.71 (1H, t, *J* = 7.5 Hz), 7.52 (1H, d, *J* = 7.5 Hz)) (table 1). Moreover, the ^13^C-NMR data of compound **1** showed high similarity to those of selaginellin H in the literature [4] except for the absence of signals for the hydroxymethyl group, which indicated that the hydroxymethyl group was degraded in **1**. This was supported by the ^1^H–^1^H COSY correlations (H15/H16 and H16/H17) of A-ring and detailed 2D analysis (figure 2).

**Figure 3.**
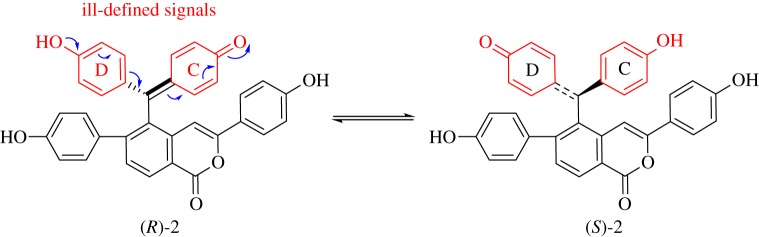
Tautomerization of compound **2** resulting in ill-defined signals of CD rings.

**Scheme 1.**
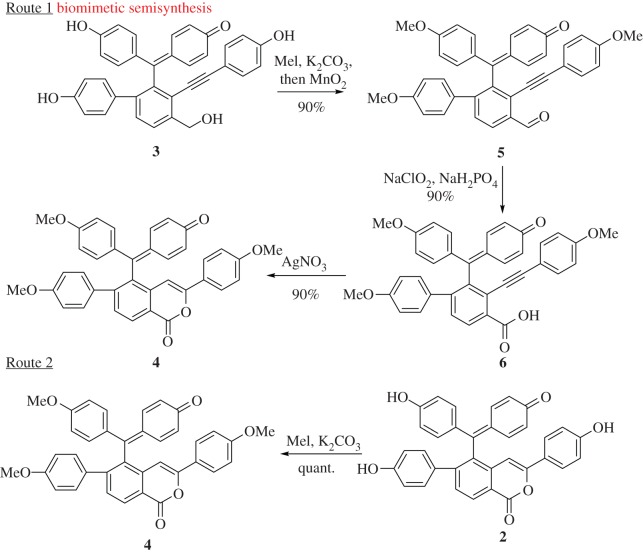
Cross-validated the structure of **2.**

Selaginellin U (**2**) was obtained as a yellow oil, and showed ion peaks at *m/z* 525.13372 [M − H]^−^ (calcd 525.1344) in HR-ESI-MS analysis corresponding to the molecular formula C_34_H_22_O_6_. The ^1^H and ^13^C-NMR spectra of **2** displayed an AB spin system (*δ*_H_ 7.54 and 8.36, each 1H, d, *J* = 8.1 Hz) for the *ortho*-tetrasubstituted A-ring, two AA'BB’ systems for the respective *para*-disubstituted B- and E-rings ((*δ*_H_ 6.88 and 7.58, each 2H,d, *J* = 8.5 Hz), (*δ*_H_ 6.69 and 6.97, each 2H,d, *J* = 8.4 Hz)), and an olefinic hydrogen (*δ*_H_ 6.71, s). C26 was supposed to link to C-15 by HMBC correlation from H16 to C26 (figure 2). Strong HMBC correlations from H27 to C15 and C19 revealed that C27 linked to C14. The B-ring was determined to link to C28 by HMBC correlation from H33 to C28. The down-fielded C28 and up-fielded C15, as well as the single signal of H27 revealed that C26 and C28 probably were connected through an ester bond, resulting in the formation of a possibly new ring F fused with ring A at the C14 and C15 positions. However, the structural elucidation was stopped here because of the mismatching of the number of atoms present (C_22_H_13_O_4_) with the molecular formula C_34_H_22_O_6__,_ which resulted from HRMS. Selaginellins with *p*-benzoquinone-linked *p*-phenol motif always existed in two tautomers [2], resulting in delocalization between the C- and D-ring and rotation of the C7–C19 bond, and consequent broadening signals of CD rings, part of them even appearing to be obscured in the baseline (figure 3). Subsequently, attempts to render well-defined NMR signals by acquiring the NMR spectra in a different solvent CD_3_OD (insoluble in other solvents) or with changing the test temperature (0 °C and 40 °C) failed. Therefore, a biomimetic semisynthesis of trimethyl ether **4** from selaginellin (**3**) was carried out for confirming the existence of CD rings to draw the whole structural picture of compound **2** (scheme 1).

As outlined in scheme 1, chemoselective methylation of three phenol hydroxyl groups of **3** under the condition of MeI/K_2_CO_3_ and subsequent MnO_2_ oxidation gave methylated aldehyde **5** in a 90% yield over two steps. Next, compound **5** was converted to acid **6** through pinnic oxidation in a 90% yield [12]. According to the hypothetical biogenetic pathway of **2** (electronic supplementary material, scheme S1), the end game was a biomimetic intramolecular cyclization of **6** in the presence of catalytic AgNO_3_ which furnished cyclic trimethyl ether **4** in a 90% yield. To validate that **4** bears identical structure to natural **2**, compound **2** was subsequently fully methylated using MeI/K_2_CO_3_ to give the desired product **4** in a quantitative yield.

As expected, all overlapped signals were well resolved in the NMR spectra of compounds **4**, **5** and **6**. The tautomerization between phenol and benzoquinone at CD rings was blocked by methylation of three phenol hydroxyl groups successfully. The NMR data of compound **4** obtained from route 1 was coincident with those from route 2 (tables 1 and 2**, Δ*δ*** ≤ 0.01 ppm in ^1^H NMR and ≤ 0.1 ppm in ^13^C NMR). Thus, selaginellin was probably regarded as the biogenetic precursor of **2**.

Compound **4** is a yellow oil and its molecular formula C_37_H_28_O_6_ was determined by HR-ESI-MS at *m/z* 607.1517 [M + K]^+^ (calcd *m/z* 607.1517). Compared to the ^1^H-NMR spectra of **2**, another *p*-phenyl group (*δ*_H_ 6.97 (2H, d, *J* = 8.8 Hz), 6.82 (2H, d, *J* = 8.8 Hz)) representing the *para*-substituted D-ring proton could be found in the spectra of **4**, as well as three methoxyl groups (*δ*_H_ 3.76 (3H, s), 3.78 (3H, s), 3.82 (3H, s)) (table 1). The signals at *δ*_H_ 7.59 (1H, dd, *J* = 10.0, 2.7 Hz), 7.23 (1H, dd, *J* = 10.0, 2.7 Hz), 6.38 (1H, dd, *J* = 10.0, 2.7 Hz) and 6.27 (1H, dd, *J* = 10.0, 2.7 Hz) represent D-ring protons. Further analysis of the ^13^C-NMR and DEPT of compound **4** revealed a carbonyl group (*δ*_C_ = 186.5) (table 2). ^1^H–^1^H COSY correlations (H2/H3, H5/H6) and HMBC correlations (H3, H5/C1; H2, H6/C4) implied a semi-quinone unit (C-ring) (figure 2). HMBC correlations (H3/C7, H5/C7; H8, H12/C7) indicated that both C- and D-rings were connected via C7 (*δ*_C_ = 155.1). The E-ring was found connected to the A-ring at C-18 (*δ*_C_ = 149.4) via HMBC correlations (H20, H24/C18; H2, H17/C25). The B-ring was linked to C28 by HMBC correlation of H32/C27. Subsequently, C19 was the last position left for C7 at ring A. Thus, by comparison with analysis of the structure of compound **4**, the structure of compound **2** was established as 3,6-bis(4-hydroxyphenyl)-5-((4-hydroxyphenyl)(4-oxocyclohexa-2,5-dien-1-ylidene)methyl)-1H-isochromen-1-one, named selaginellin U.

The known compounds were identified as selaginpulvilin A (**7**) [8], selaginpulvilin B (**8**) [8], selaginpulvilin C (**9**) [8], selaginpulvilin D (**10**) [8], selaginpulvilin E (**11**) [11], selaginellin (**3**) [2], selaginellin A (**12**) [13], selaginellin B (**13**) [13], selaginellin G (**14**) [4], selaginellin M (**15**) [5] and selaginellin O (**16**) [7] by comparing their spectroscopic and physical data with those published.

All natural compounds were tested for their inhibitory activity against BACE1; compounds **3**, **13** and **16** possessed good inhibitory activity against BACE1 (IC_50_ = 81.17, 51.13 and 48.89 µM, respectively) (electronic supplementary material, table S1).

## 4. Conclusion

In conclusion, two new lactone-containing selaginellins **1** and **2**, together with eleven known ones, were isolated from *S. tamariscina*. Among them, selaginellin U (**2**), with a rare unsaturated *δ*-lactone ring fused with ring A, represents a new structure type for selaginellins. In addition, we first carried out biomimetic semisynthesis of compound **4**, which not only enabled structural elucidation of **2** but also elucidated the plausible mechanisms of its biogenetic pathway. Furthermore, compounds **3**, **13** and **16** exhibited promising application in the treatment of Alzheimer's disease owing to their good inhibitory activities against BACE1.

## Supplementary Material

Biogenetic pathway, bioassay, and NMR copies

## Data accessibility

The dataset supporting this article has been uploaded as the electronic supplementary material (https://doi.org/10.6084/m9.figshare.5104462.v1).
