# Supplementary material for: A biomimetic semisynthesis enables structural elucidation of selaginellin U: a tautomeric cyclic alkynylphenol from Selaginella tamariscina
Source: R Soc Open Sci. 2017 Jul 19;4(7):170352. doi: 10.1098/rsos.170352 (PMC5541560; doi:10.1098/rsos.170352)

A Biomimetic Semisynthesis Enables Structural Elucidation of Selaginellin U: A Tautomeric Cyclic Alkynylphenol from *Selaginella tamariscina*

Qin-Feng Zhu, ^1,2^ Ying Bao, ^1,2^ Zhi-Jun Zhang, ^1,2^ Jia Su, ^1^ Li-Dong Shao, ^1^ and Qin-Shi Zhao ^1^

*^1^ State Key Laboratory of Phytochemistry and Plant Resources in West China, Kunming Institute of Botany, Chinese Academy of Sciences, Kunming 650201, People’s Republic of China*

*^2^University of Chinese Academy of Sciences, Beijing 100049, People’s Republic of China*

**Table of Contents**

**Scheme S1.** Hypothetical biogenetic pathway of compounds **1** and **2………………………………………**S2

**Table S1.** Inhibitory activities (IC_50_, *μ*M) of Compounds **1**-**3** and **7**-**16** against the BACE1…S3

**Figure S2.-S7.** NMR spectrum of selaginellin T (**1**)…………………………………………………………….S4

**Figure S8.** HR ESI MS spectrum of selaginellin T (**1**)………………………………………………………….S7

**Figure S9.** UV spectrum of selaginellin T (**1**)……………………………………………………………………..S8

**Figure S10.** IR spectrum of selaginellin T (**1**)……………………………………………………………………..S8

**Figure S11.-S15.** NMR spectrum of selaginellin U (**2**)………………………………………………………..S9

**Figure S16.** HR ESI MS spectrum of selaginellin U (**2**)………………………………….………………….S11

**Figure S17.** UV spectrum of selaginellin U (**2**)…………………………………………………………………S12

**Figure S18.** IR spectrum of selaginellin U (**2**)…………………………………………………………………..S12

**Figure S19.-S20.** NMR spectrum of selaginellin (**3**)………………………………………………………….S13

**Figure S21.1-S26.** NMR spectrum of compound (**4**)…………………………………………………………S14

**Figure S27.** HR ESI MS spectrum of compound (**4**)………………………………………………………….S18

**Figure S28-S29.** NMR spectrum of compound (**5**)……………………………………………………………S18

**Figure S30.** HR ESI MS spectrum of compound (**5**)………………………………………………………….S19

**Figure S31-32.** NMR spectrum of compound (**6**)…………………………………….………………………S20

**Figure S33.** HR ESI MS spectrum of compound (**6**)………………………………………………………….S21

**Figure S34.-S35.** NMR spectrum of selaginpulvilin A (**7**)……………………..…………………………….S22

**Figure S36.-S37.** NMR spectrum of selaginpulvilin B (**8**)……………………………………………………S23

**Figure S38.-S39.**NMR spectrum of selaginpulvilin C (**9**)…………………………………………………….S24

**Figure S40.-S41.** NMR spectrum of selaginpulvilin D (**10**)………………………………………………….S25

**Figure S42.-S46.** NMR spectrum of selaginpulvilin E (**11**)………………………………………….………S26

**Figure S47.-S48.** NMR spectrum of selaginellin A (**12**)……………………………………………..……..S29

**Figure S49.-S50.** NMR spectrum of selaginellin B (**13**)………………………………………….…………S30

**Figure S51.-S52.** NMR spectrum of selaginellin G (**14**)…………………………………………………….S31

**Figure S53.-S54.** NMR spectrum of selaginellin M (**15**)……………………………………………………S32

**Figure S55.-S56.** NMR spectrum of selaginellin O (**16**)…………………………………………………….S33

**Scheme S1.** Hypothetical biogenetic pathway of compounds **1** and **2**

**Table S1. Inhibitory activities (IC_50_, *μ*M) of compounds 1-3 and 7-16 against the BACE1**

| **Compounds** | **IC_50_** | **Compounds** | **IC_50_** |
| --- | --- | --- | --- |
| **1** | >100 | **11** | >100 |
| **2** | >100 | **12** | >100 |
| **3** | 81.17 | **13** | 51.13 |
| **7** | >100 | **14** | >100 |
| **8** | >100 | **15** | >100 |
| **9** | >100 | **16** | 48.89 |
| **10** | >100 | LY2811376 | 392nM |

Figure **S2.** ^1^H NMR spectrum of selaginellin T (1) in acetone-d_6_


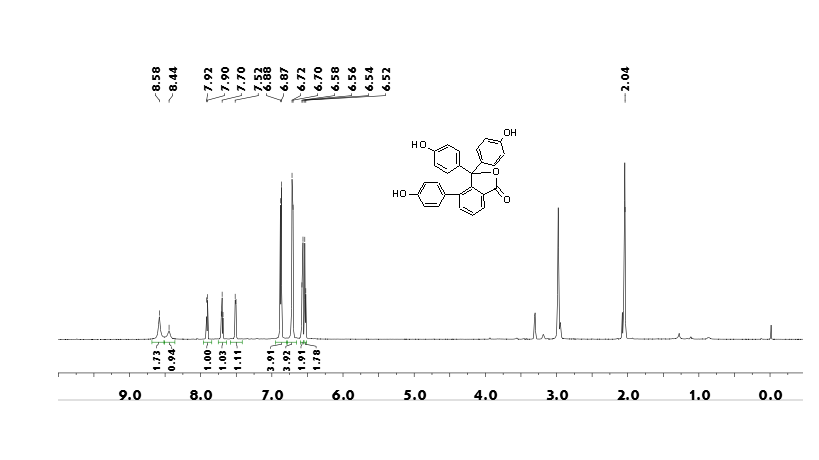


Figure **S3**. ^13^C NMR spectrum of selaginellin T (1) in acetone-d_6_


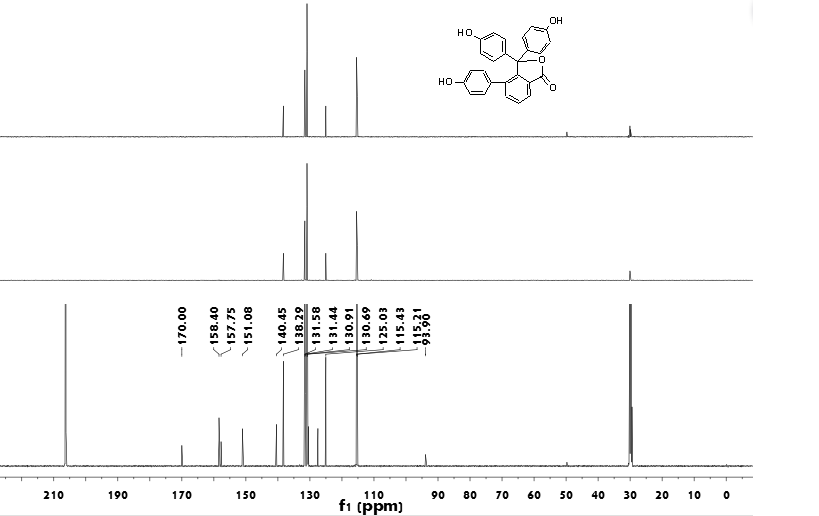


Figure **S4.** ^1^H-^1^H COSY spectrum of selaginellin T (1) in acetone-d_6_


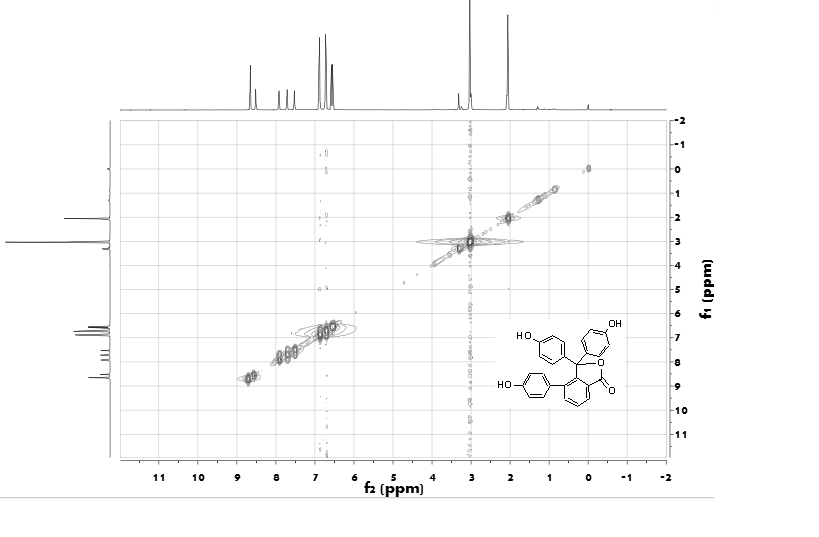


Figure **S5.** HSQC spectrum of selaginellin T (1) in acetone-d_6_


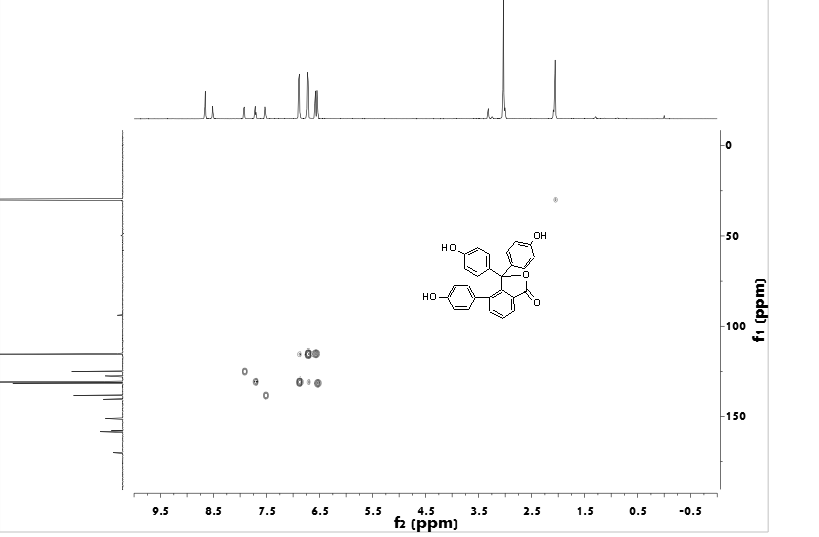


Figure **S6.** HMBC spectrum of selaginellin T (1) in acetone-d_6_


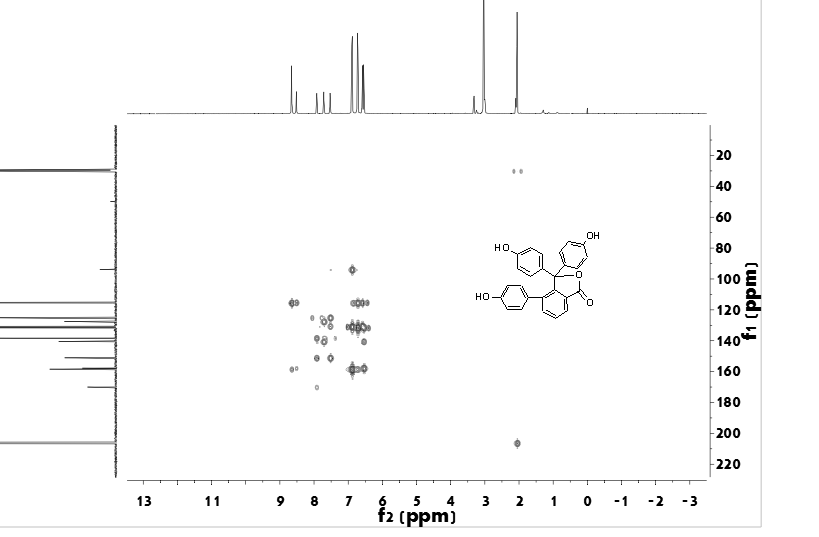


Figure **S7.** ROESY spectrum of selaginellin T (1) in acetone-d_6_


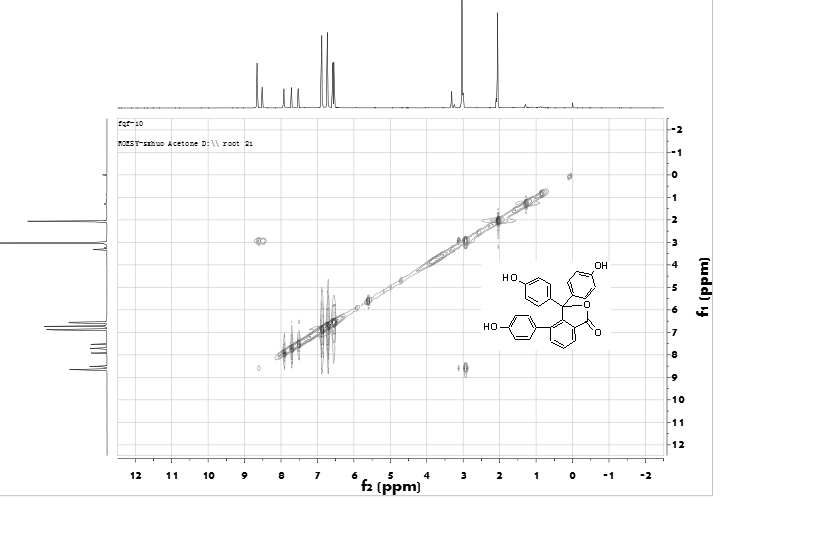


Figure **S8.** HR ESI MS spectrum of selaginellin T (1)


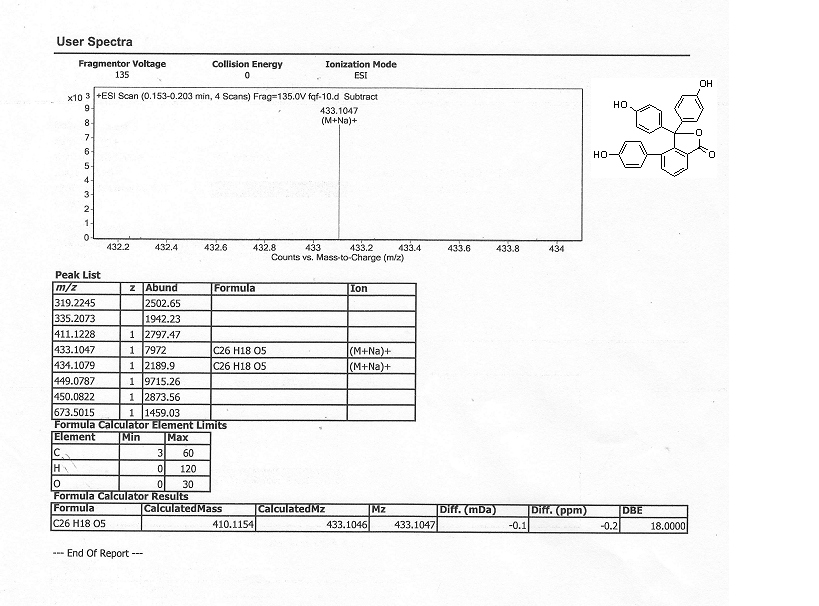


Figure **S9.** UV spectrum of selaginellin T (1)


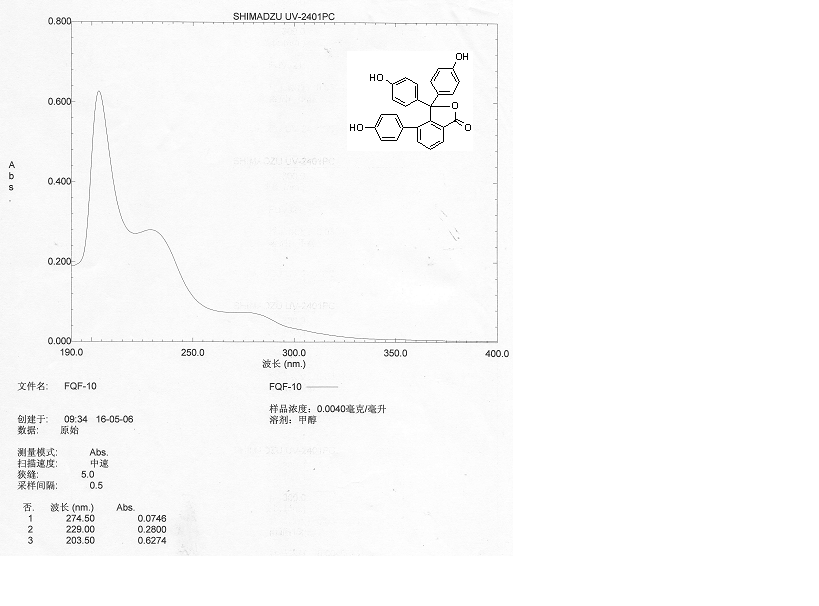


Figure **S10.** IR spectrum of selaginellin T (1)


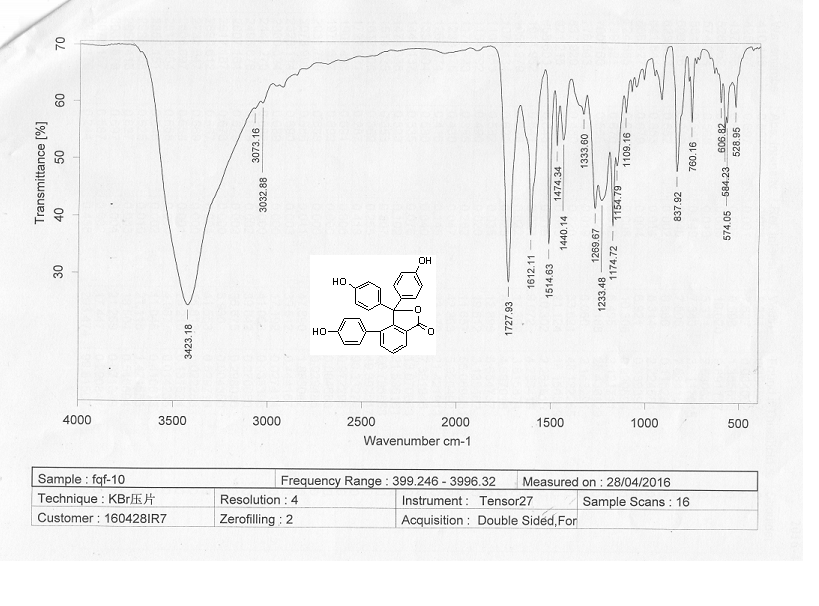


Figure **S11.** ^1^H NMR spectrum of selaginellin U (2) in acetone-d_6_


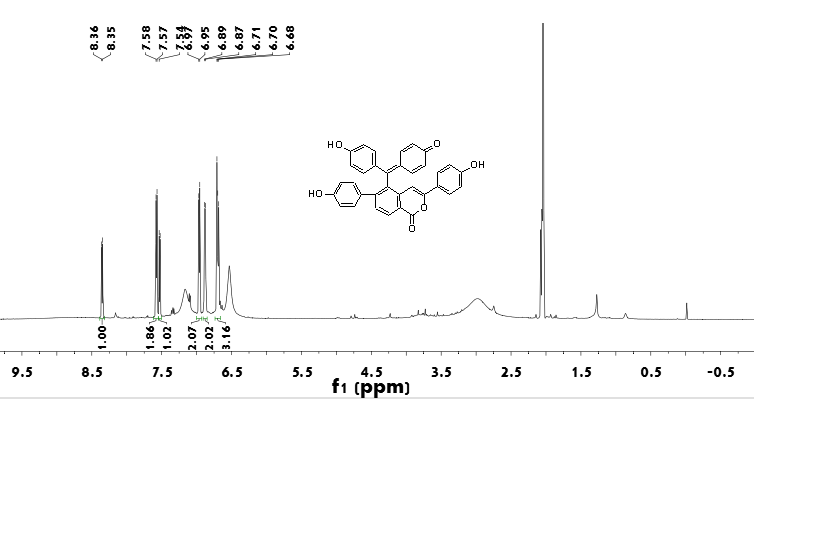


Figure **S12**. ^13^C NMR spectrum of selaginellin U (2) in acetone-d_6_


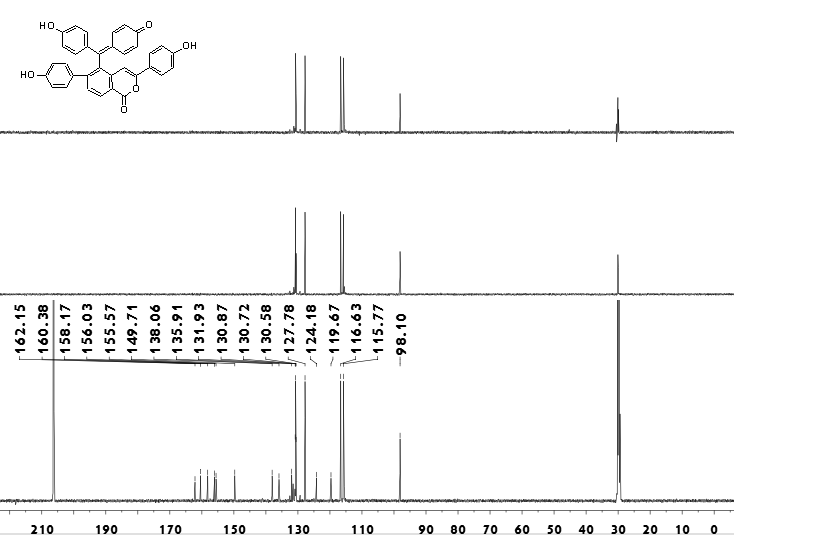


Figure **S13.** ^1^H-^1^H COSY spectrum of selaginellin U (2) in acetone-d_6_


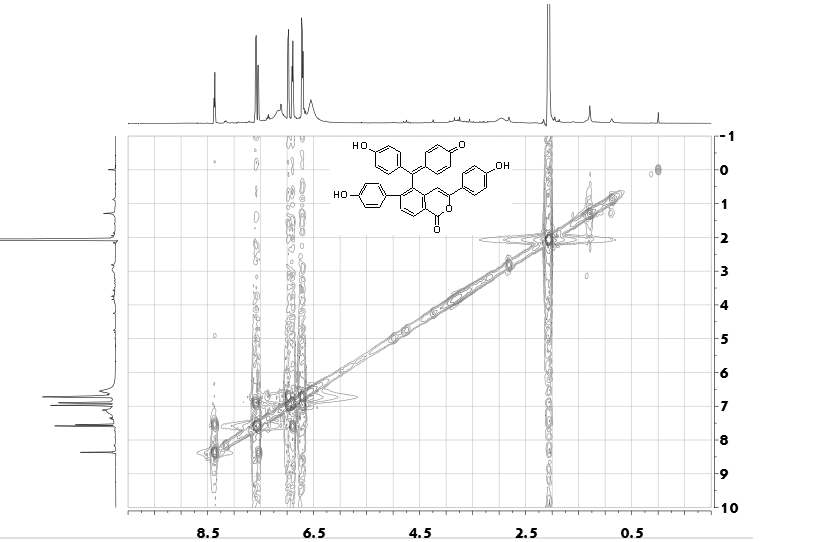


Figure **S14.** HSQC spectrum of selaginellin U (2) in acetone-d_6_


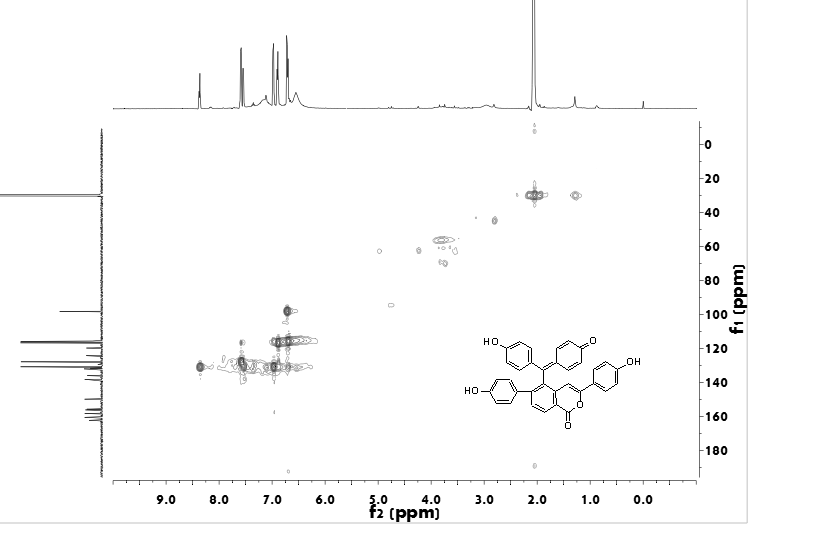


Figure **S15.** HMBC spectrum of selaginellin U (2) in acetone-d_6_


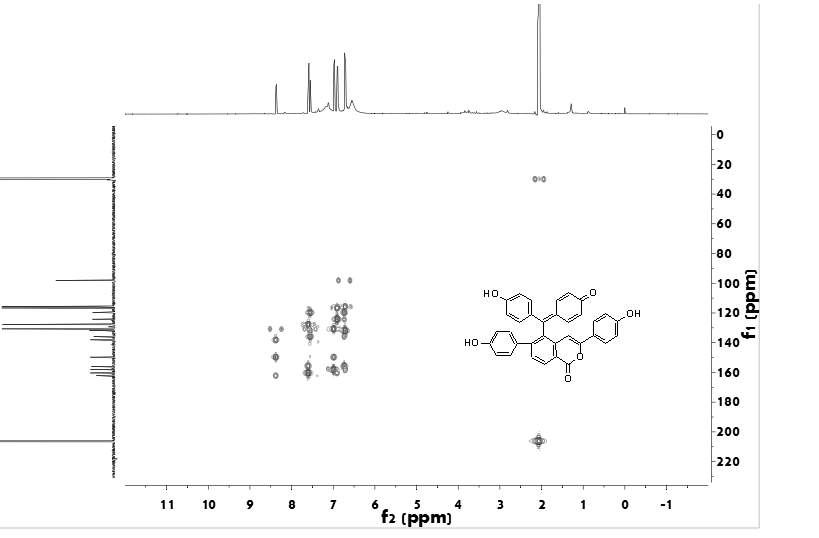


Figure **S16.** HR ESI MS spectrum of selaginellin U (2)


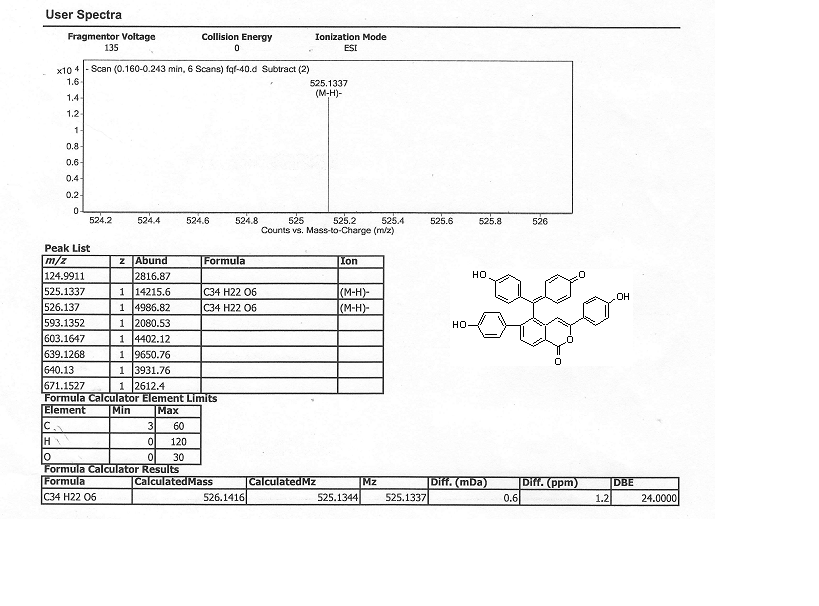


Figure **S17.** UV spectrum of selaginellin U (2)


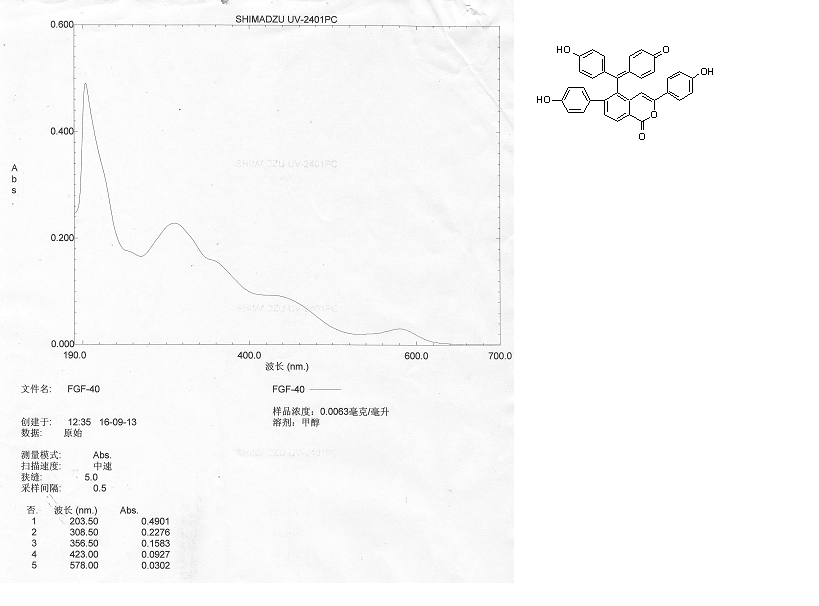


Figure **S18.** IR spectrum of selaginellin U (2)


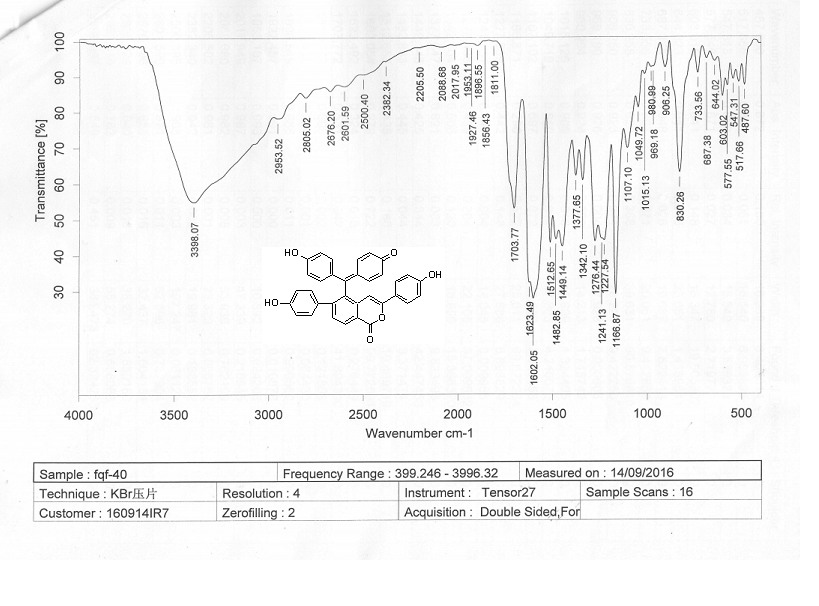


Figure **S19.** ^1^H NMR spectrum of selaginellin (3) in CD_3_OD


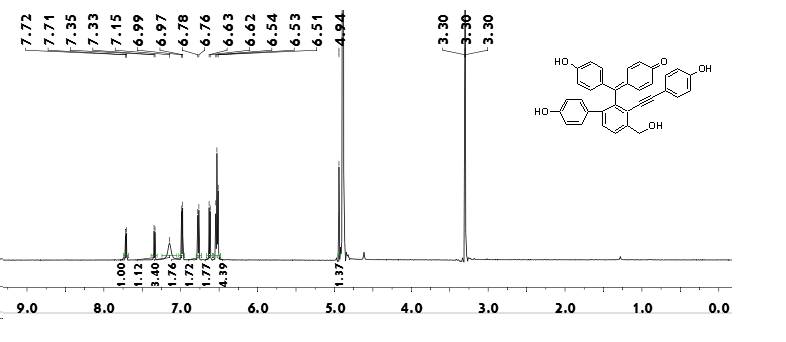


Figure **S20**. ^13^C NMR spectrum of selaginellin (3) in CD_3_OD


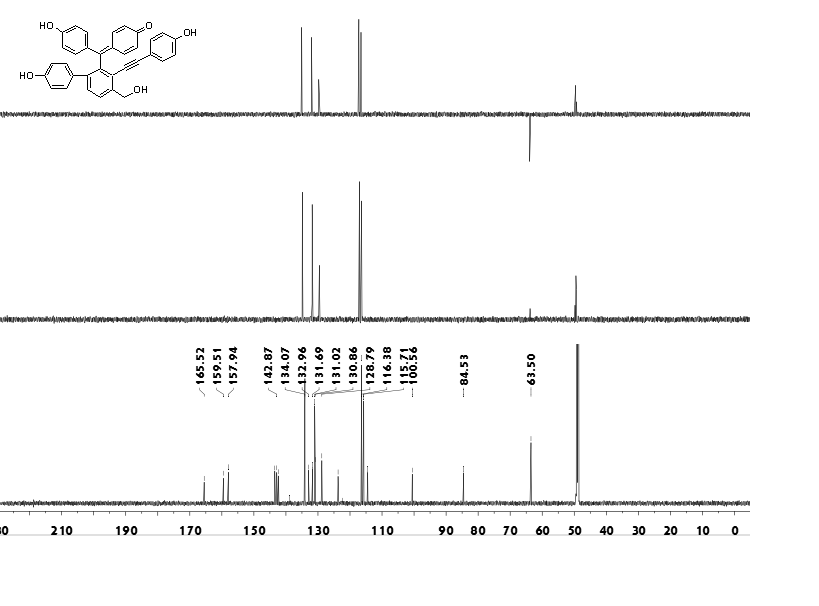


Figure **S21-1.** ^1^H NMR spectrum of compound (4) in acetone-d_6_(route 1)
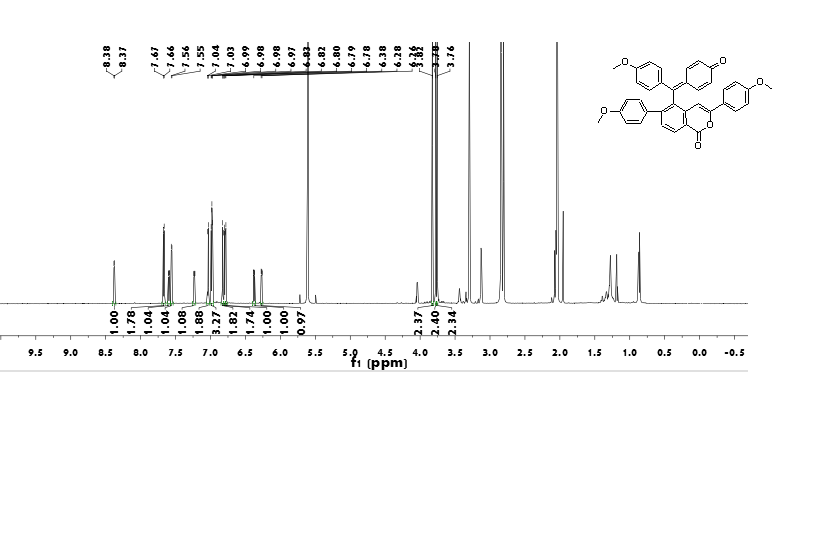


Figure **S21-2.** ^1^H NMR spectrum of compound (4) in acetone-d_6_(route 2)


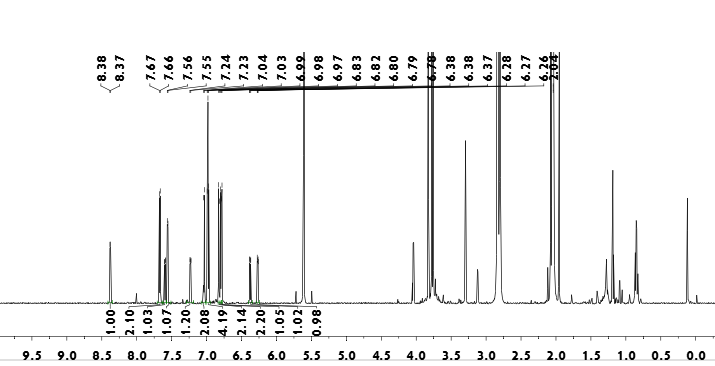


Figure **S22-1**. ^13^C NMR spectrum of compound (4) in acetone-d_6_ (route 1)


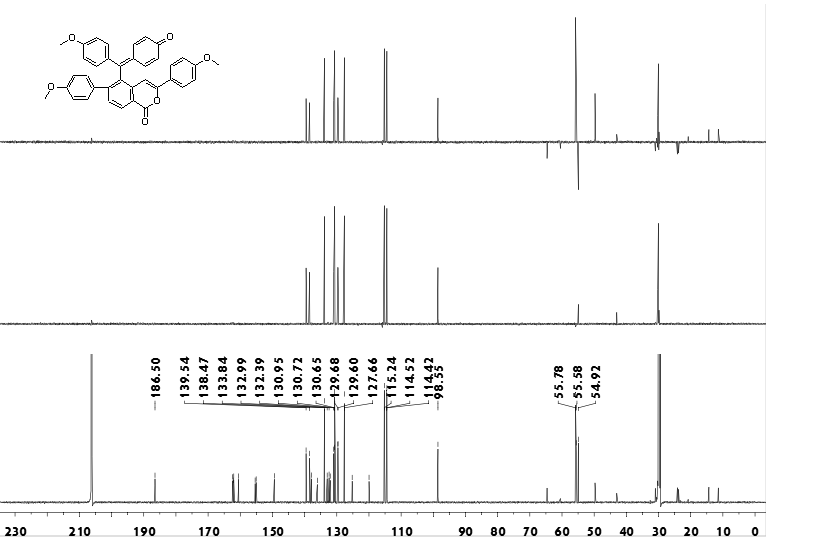


Figure **S22-2**. ^13^C NMR spectrum of compound (4) in acetone-d_6_ (route 2)


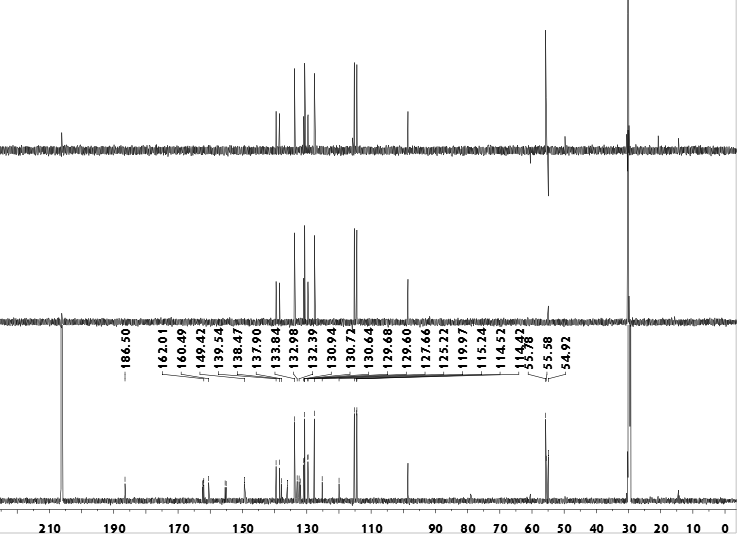


Figure **S23.** ^1^H-^1^H COSY spectrum of compound (4) in acetone-d_6_


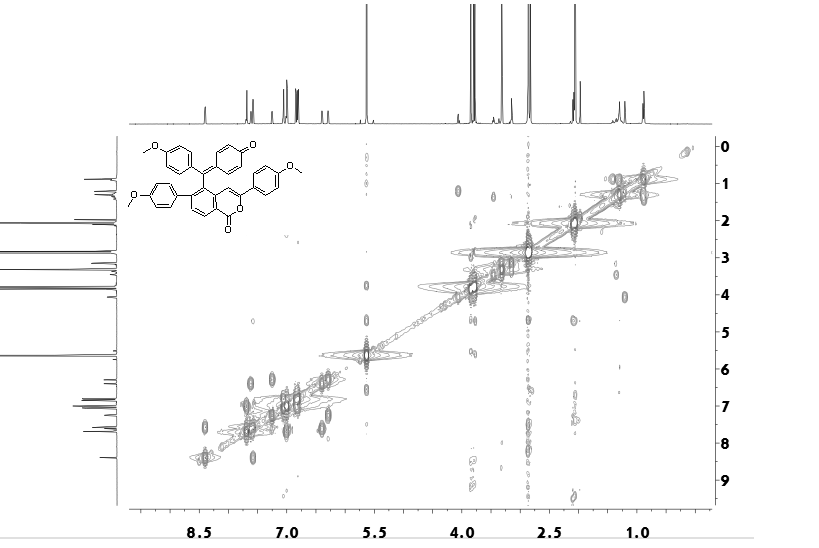


Figure **S24.** HSQC spectrum of compound (4) in acetone-d_6_


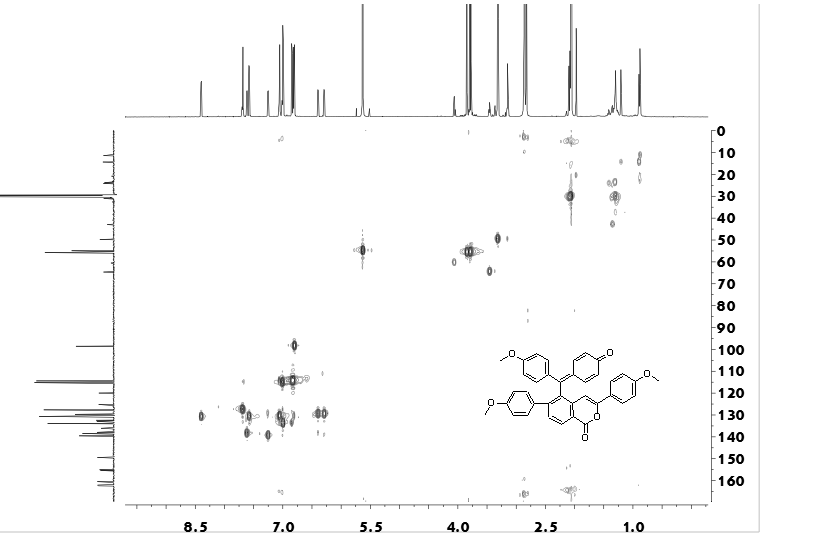


Figure **S25.** HMBC spectrum of compound (4) in acetone-d_6_


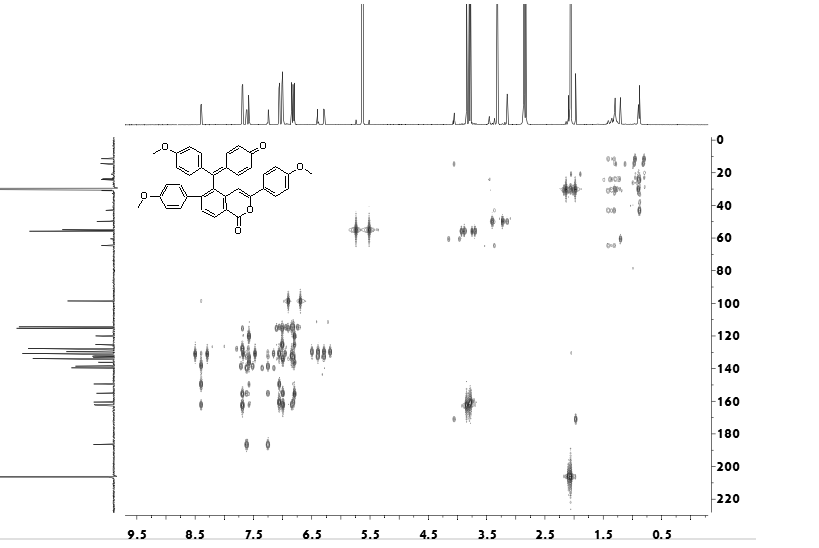


Figure **S26.** ROESY spectrum of compound (4) in acetone-d_6_


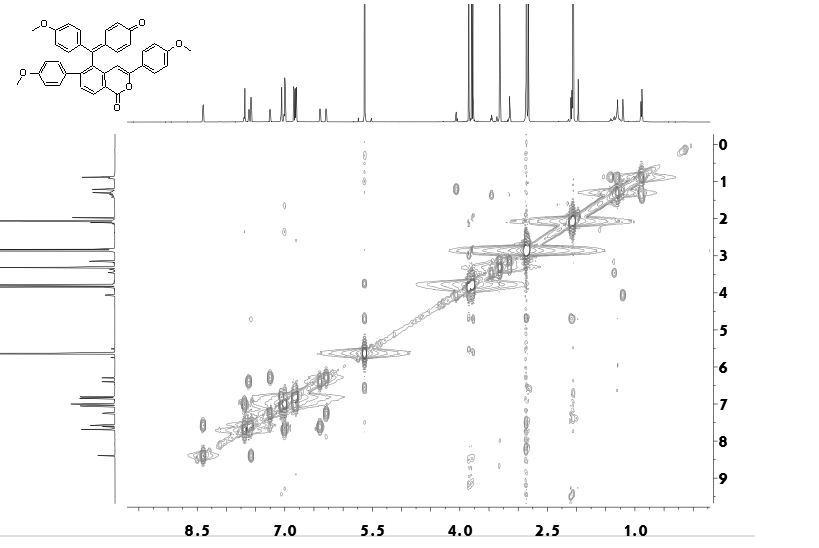


Figure **S27.** HR ESI MS spectrum of compound (4)


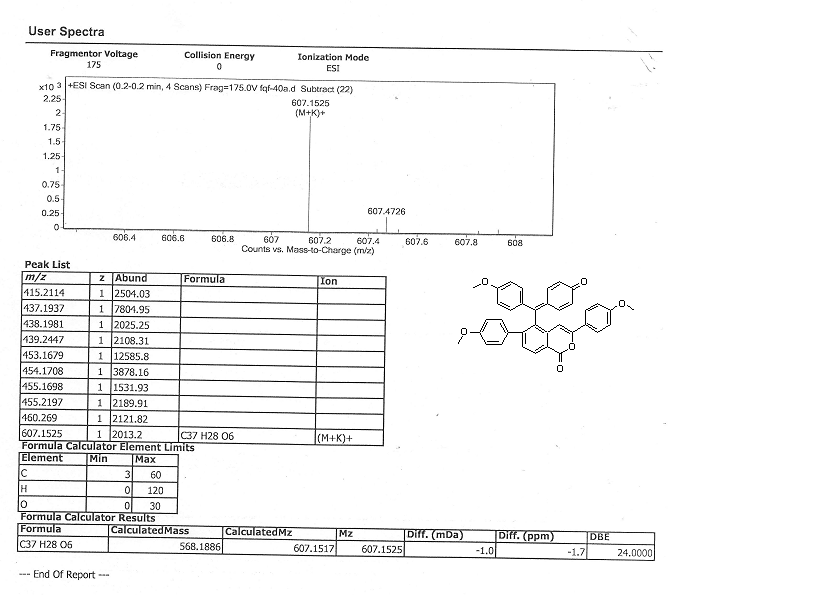


Figure **S28.** ^1^H NMR spectrum of compound (5) in acetone-d_6_


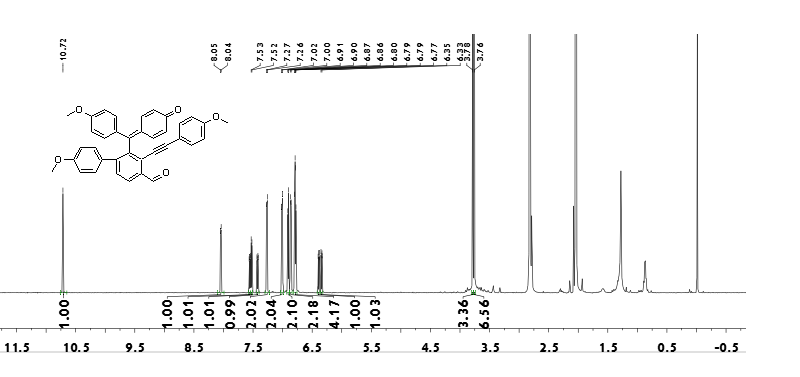


Figure **S29**. ^13^C NMR spectrum of compound (5) in acetone-d_6_


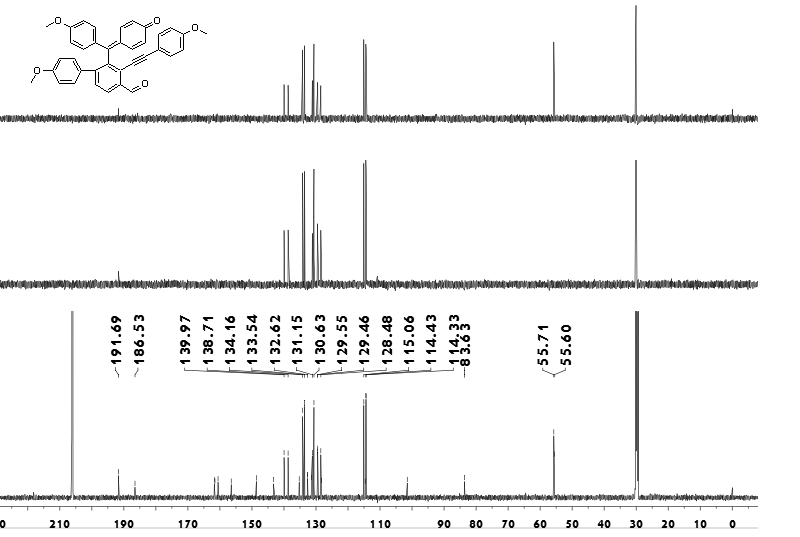


Figure **S30.** HR ESI MS spectrum of compound (5)


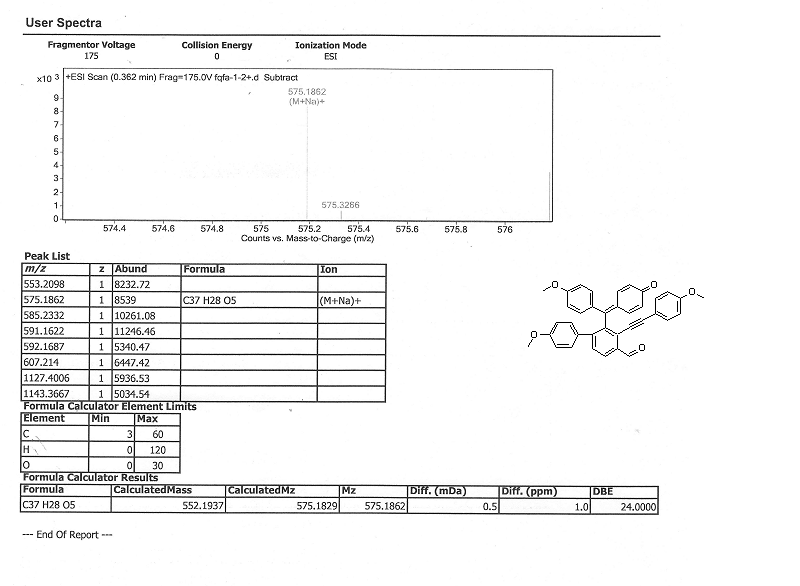


Figure **S31**. ^1^H NMR spectrum of compound (6) in DMSO-d_6_


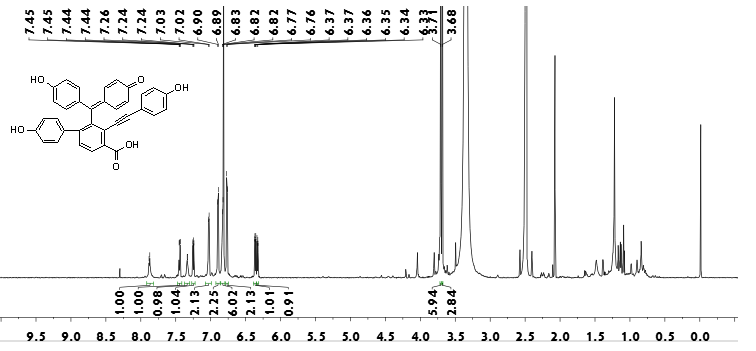


Figure **S32**. ^13^C NMR spectrum of compound (6) in DMSO-d_6_


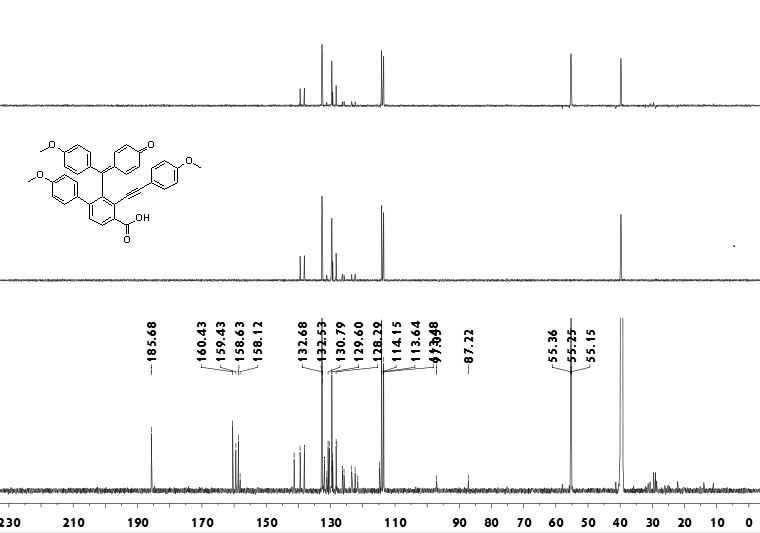


Figure **S33.** HR ESI MS spectrum of compound (6)


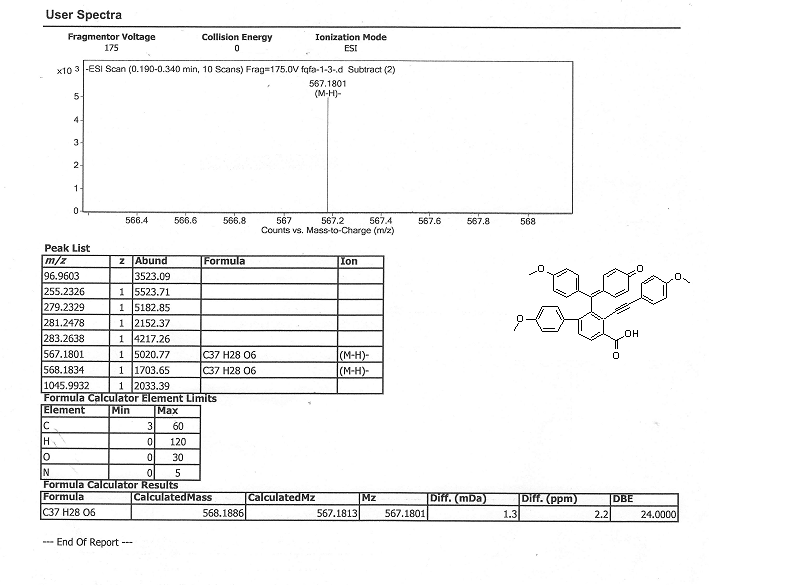


Figure **S34.** ^1^H NMR spectrum of selaginpulvilin A (7) in acetone-d_6_


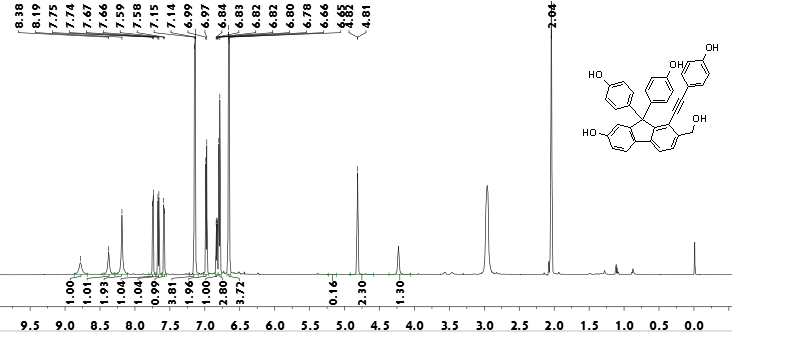


Figure **S35**. ^13^C NMR spectrum of selaginpulvilin A (7) in acetone-d_6_


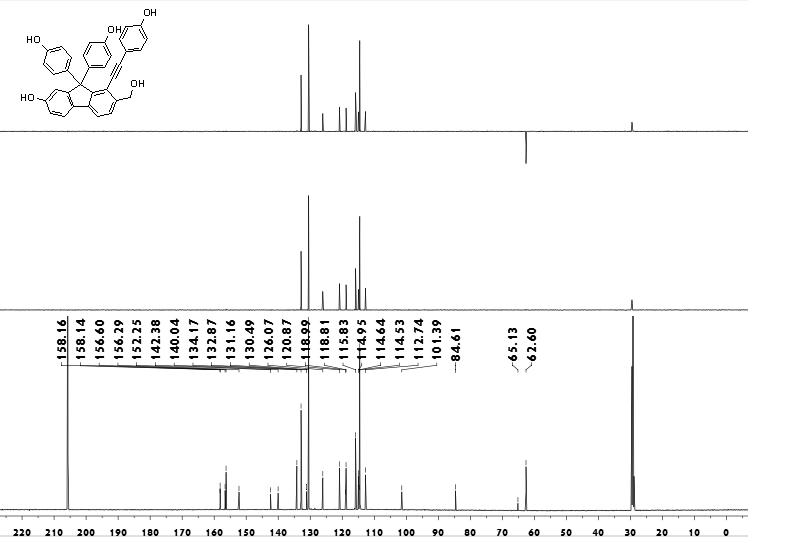


Figure **S36.** ^1^H NMR spectrum of selaginpulvilin B (8) in acetone-d_6_


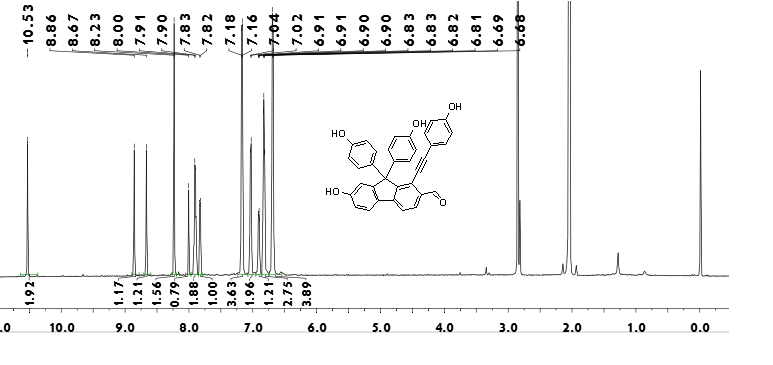


Figure **S37**. ^13^C NMR spectrum of selaginpulvilin B (8) in acetone-d_6_


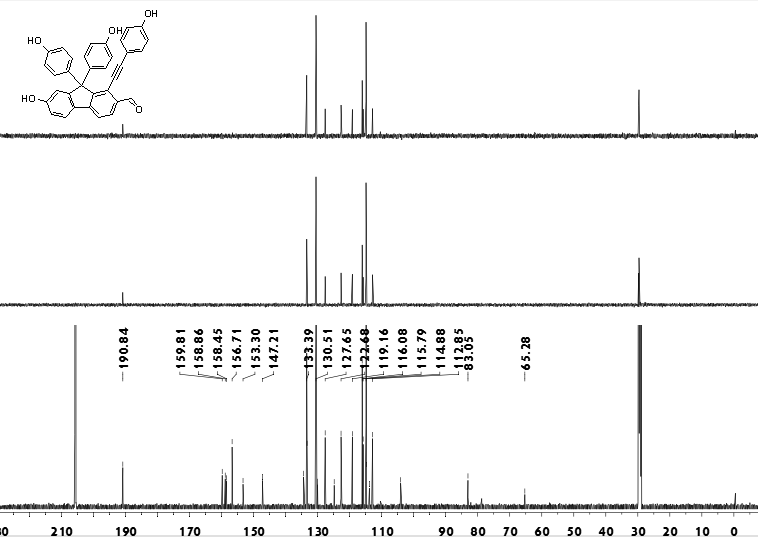


Figure **S38.** ^1^H NMR spectrum of selaginpulvilin C (9) in acetone-d_6_


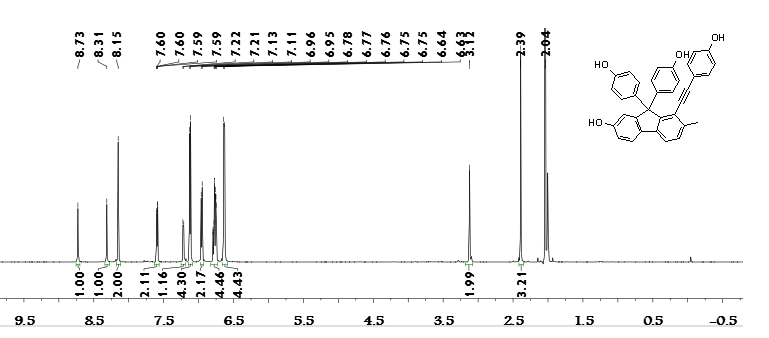


Figure **S39**. ^13^C NMR spectrum of selaginpulvilin C (9) in acetone-d_6_


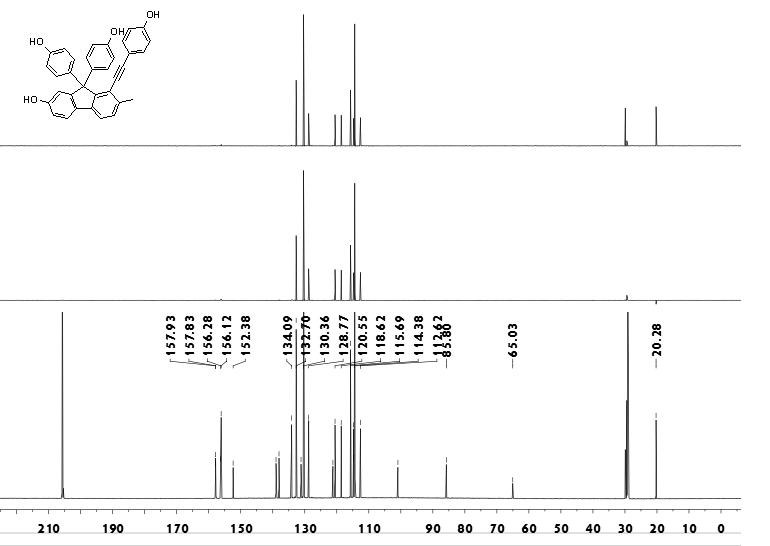


Figure **S40.** ^1^H NMR spectrum of selaginpulvilin D (10) in acetone-d_6_


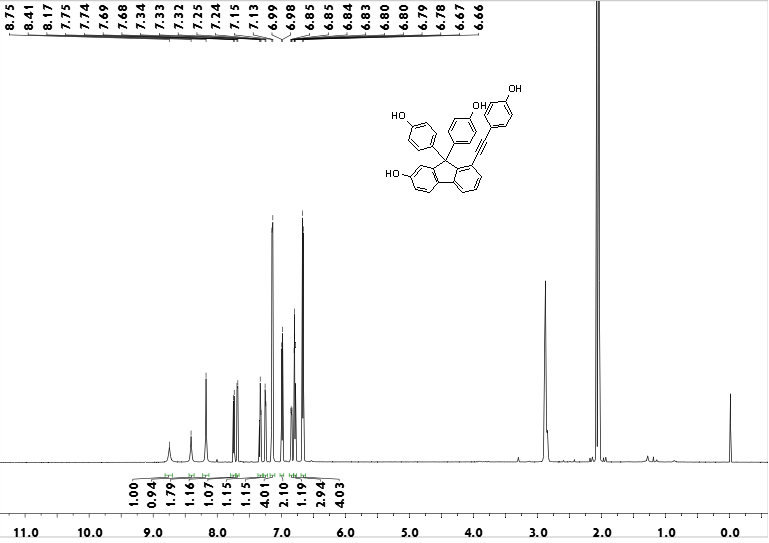


Figure **S41**. ^13^C NMR spectrum of selaginpulvilin D (10) in acetone-d_6_


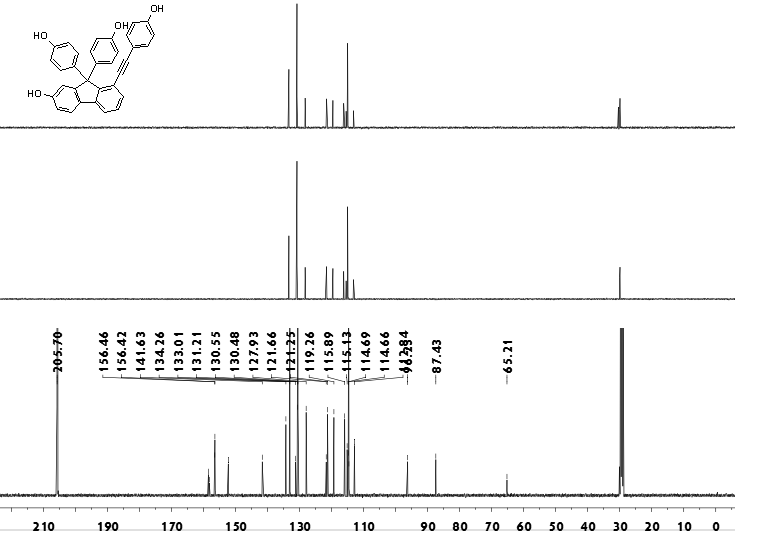


Figure **S42.** ^1^H NMR spectrum of selaginpulvilin E (11) in acetone-d_6_


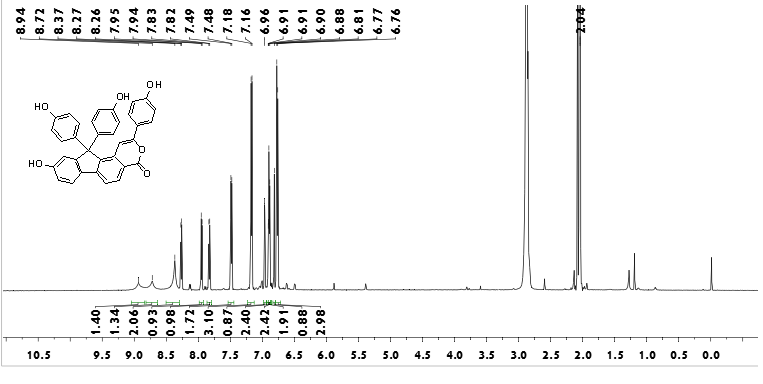


Figure **S43**. ^13^C NMR spectrum of selaginpulvilin E (11) in acetone-d_6_


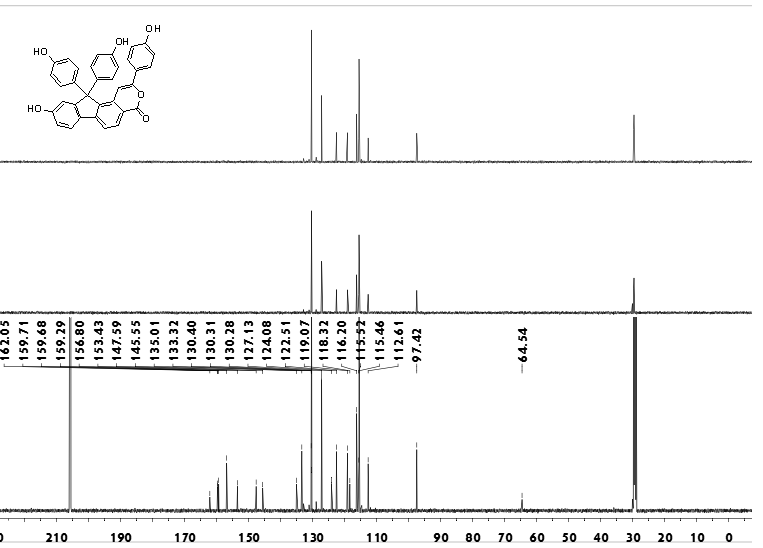


Figure **S44.** ^1^H-^1^H COSY spectrum of selaginpulvilin E (11) in acetone-d_6_


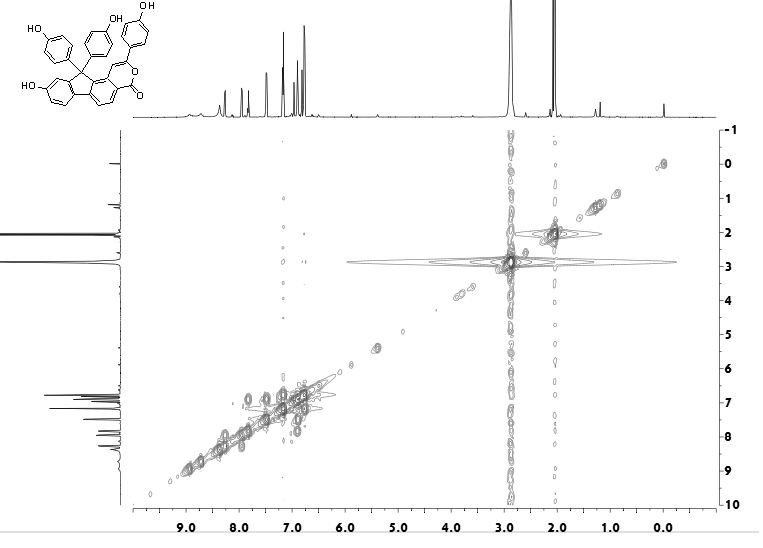


Figure **S45.** HSQC spectrum of selaginpulvilin E (11) in acetone-d_6_


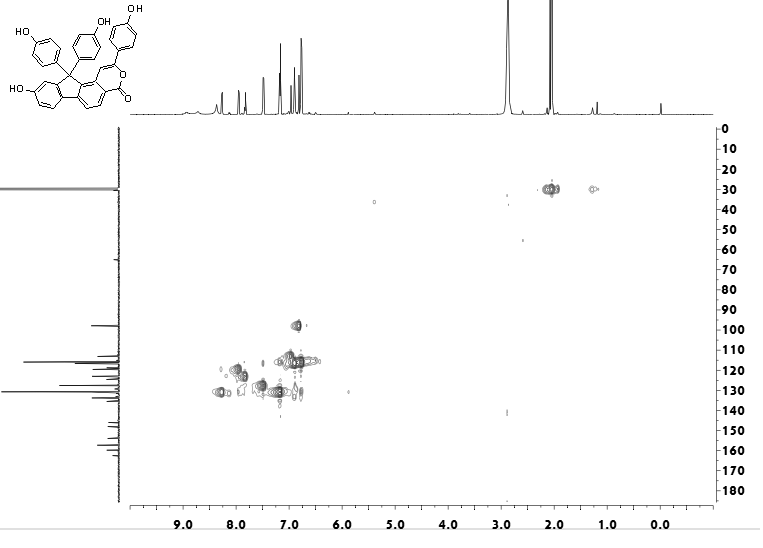


Figure **S46.** HMBC spectrum of selaginpulvilin E (11) in acetone-d_6_


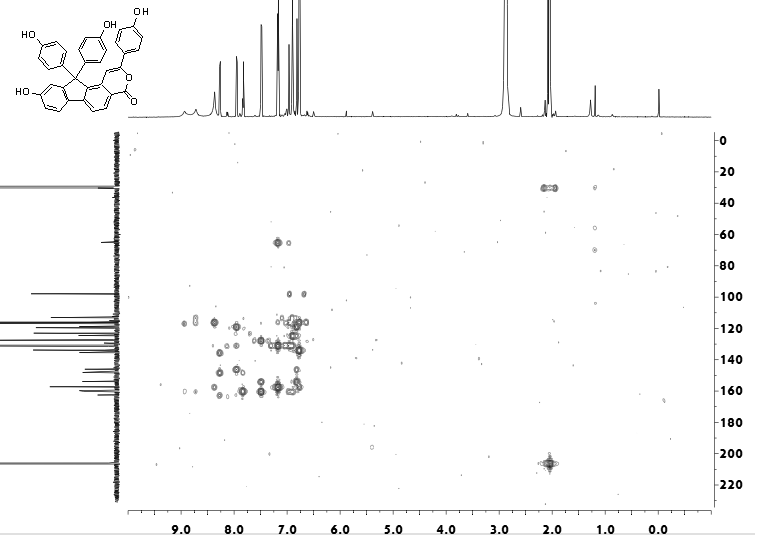


Figure **S47.** ^1^H NMR spectrum of selaginellin A (12) in CD_3_OD


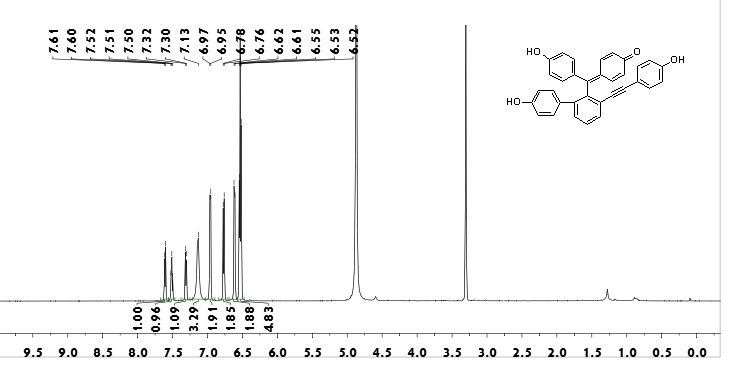


Figure **S48**. ^13^C NMR spectrum of selaginellin A (12) in CD_3_OD


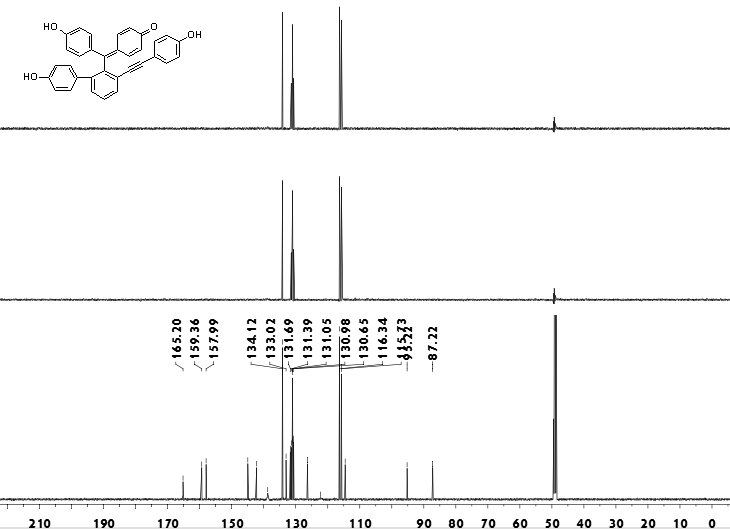


Figure **S49.** ^1^H NMR spectrum of selaginellin B (13) in acetone-d_6_


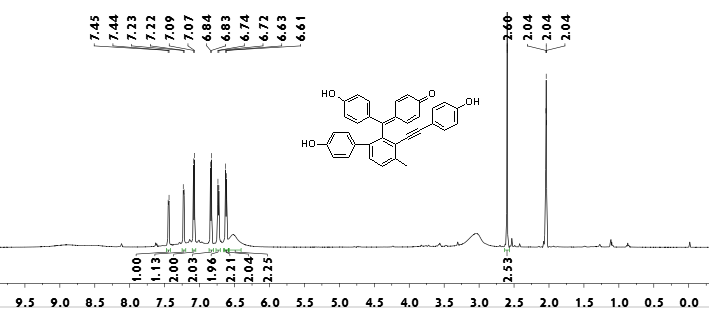


Figure **S50**. ^13^C NMR spectrum of selaginellin B (13) in acetone-d_6_


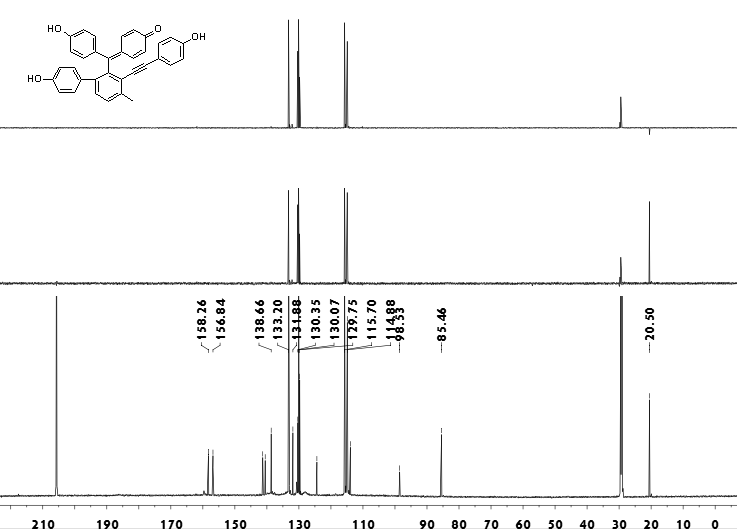


Figure **S51.** ^1^H NMR spectrum of selaginellin G (14) in acetone-d_6_


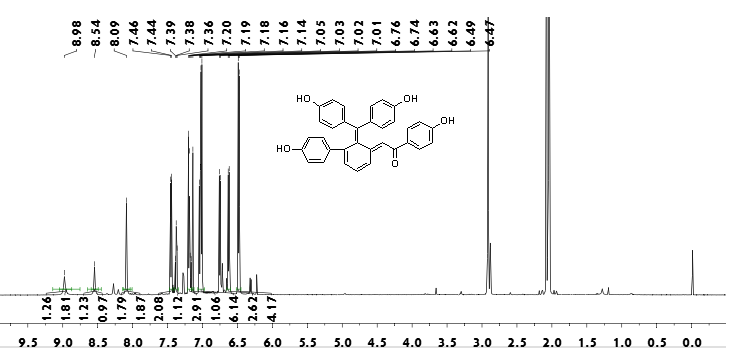


Figure **S52**. ^13^C NMR spectrum of selaginellin G (14) in acetone-d_6_


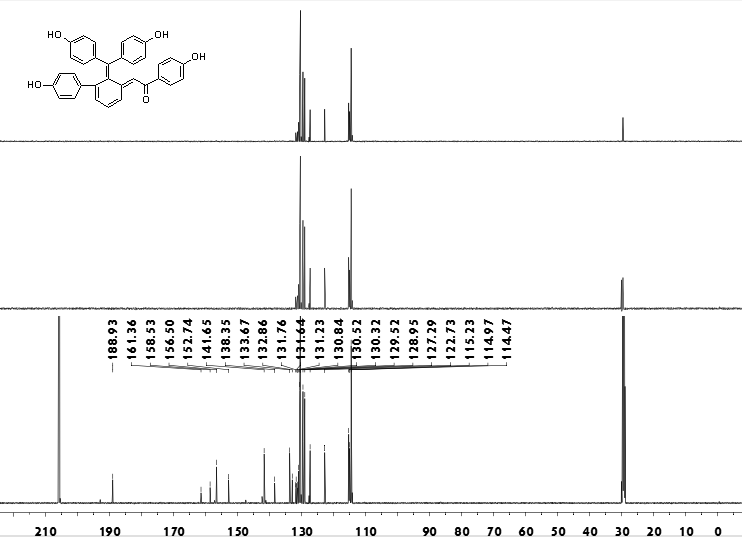


Figure **S53.** ^1^H NMR spectrum of selaginellin M (14) in acetone-d_6_


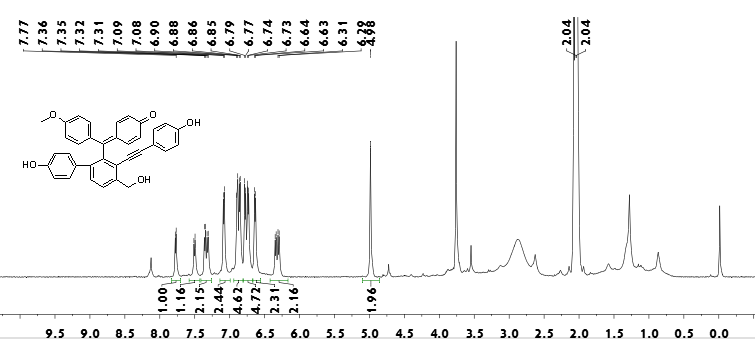


Figure **S54**. ^13^C NMR spectrum of selaginellin M (14) in acetone-d_6_


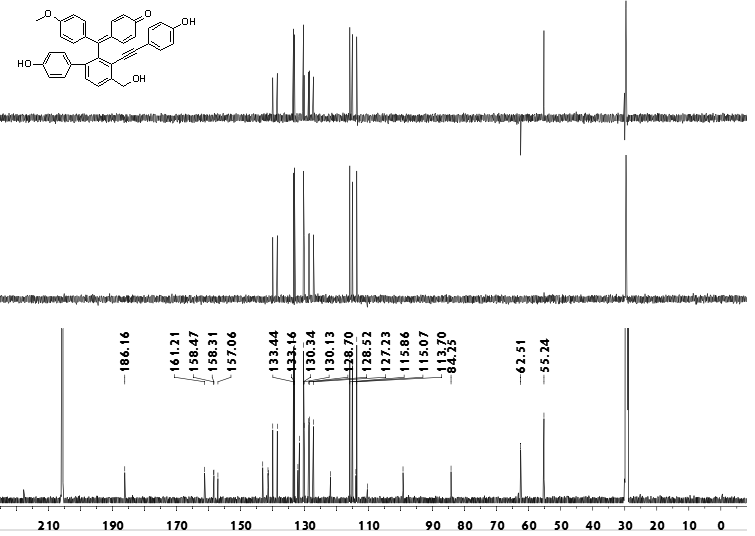


Figure **S55.** ^1^H NMR spectrum of selaginellin O (16) in acetone-d_6_


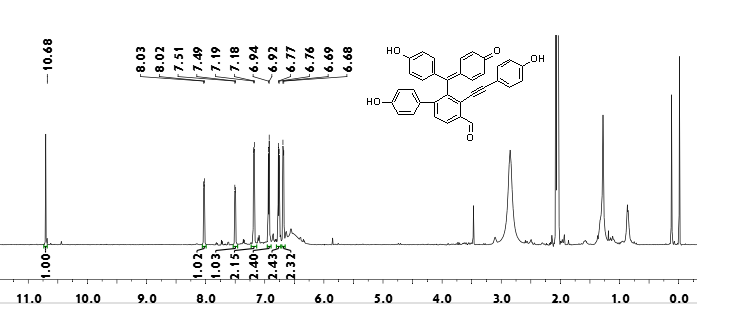


Figure **S56**. ^13^C NMR spectrum of selaginellin O (16) in acetone-d_6_


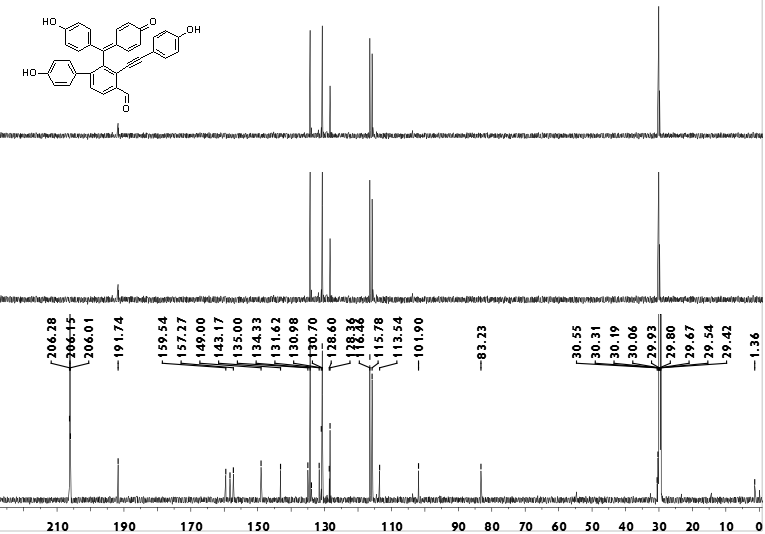

Supplement: Biogenetic pathway, bioassay, and NMR copies [file rsos170352supp1.doc]
